# 2-cyanopyridine derivatives enable N-terminal cysteine bioconjugation and peptide bond cleavage of glutathione under aqueous and mild conditions†

**DOI:** 10.1039/d4ra00437j

**Published:** 2024-02-22

**Authors:** Tetsuya Yano, Takahiro Yamada, Hiroaki Isida, Nami Ohashi, Toshimasa Itoh

**Affiliations:** a Showa Pharmaceutical University Machida Tokyo 194-8543 Japan t-yamada@ac.shoyaku.ac.jp titoh@ac.shoyaku.ac.jp

## Abstract

Inspired by the chemical reactivity of apalutamide, we have developed an efficient method for N-terminal cysteine bioconjugation with 2-cyanopyridine derivatives. Systematic investigations of various 2-cyanopyridines revealed that 2-cyanopyridines with electron-withdrawing groups react efficiently with cysteine under aqueous and mild conditions. Moreover, the highly reactive 2-cyanopyridines enable the peptide bond cleavage of glutathione. The utility of our method is demonstrated by its application to the cysteine-selective chemical modification of bioactive peptides.

## Abstract 2

Inspired by the chemical reactivity of apalutamide, an efficient method for N-terminal cysteine bioconjugation with 2-cyanopyridine derivatives has been developed.

## Graphical abstract

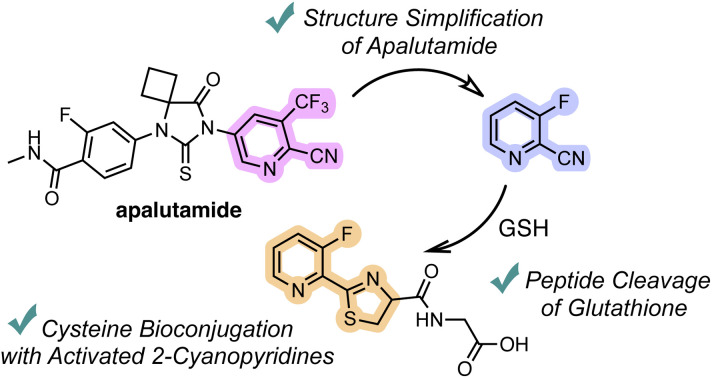

## Introduction

**Fig. 1:**
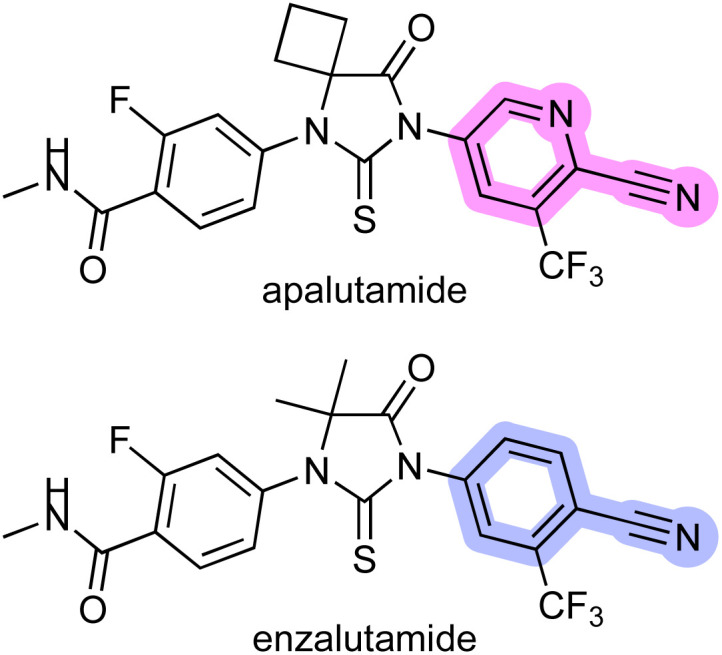
Chemical structures of apalutamide and enzalutamide.

**Scheme 1:**
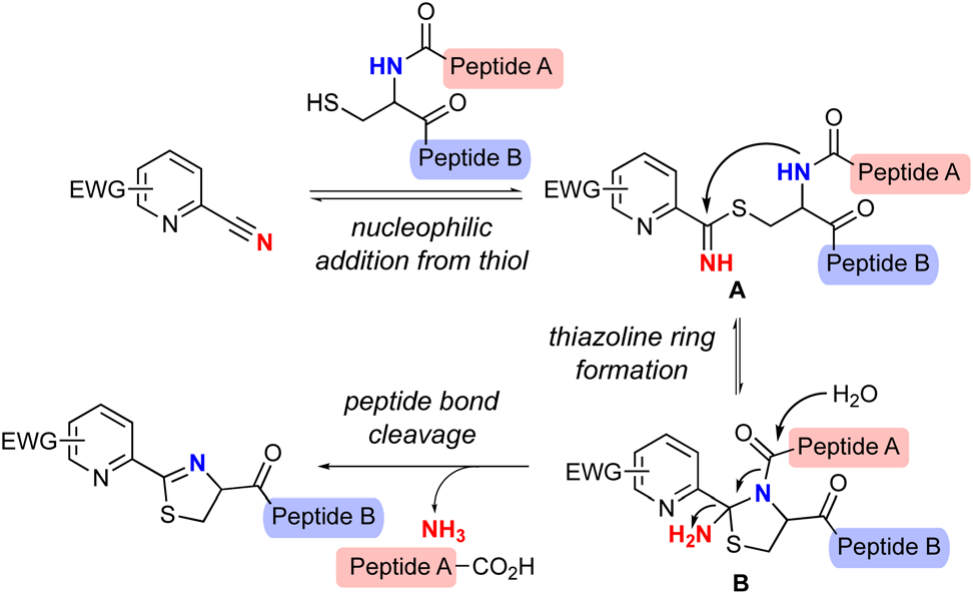
Proposed reaction mechanism of Cys-selective peptide bond cleavage by 2-cyanopyridine derivatives.

Apalutamide is a potent, specific, and orally administered inhibitor of the androgen receptor (AR) and an attractive drug for the treatment of patients with non-metastatic castration-resistant prostate cancer (nmCRPC).^1^ However, apalutamide showed a higher rate of skin rash as a side effect compared with placebo in the phase 3 SPARTAN trial.^2^ In contrast, the rate of skin rash across all clinical trials for enzalutamide,^3^ an antiandrogen drug structurally similar to apalutamide, is comparable to that of the placebo (Fig. 1).^4^ Subsequent studies suggested that the increased skin rash associated with apalutamide might be linked to a structural difference between the two drugs, and that the skin rash could be the result of drug-induced, immune-mediated hypersensitivity.^5^ The chemical structures of the two compounds show that the most important structural difference between the two drugs is that apalutamide contains a 2-cyanopyridine moiety, whereas enzalutamide possesses 2-cyanophenyl (Fig. 1). 2-Cyanopyridines have been experimentally and computationally confirmed to be more reactive than 2-cyanophenyls,^6^ and previous studies revealed that the 2-cyanopyridine moiety of apalutamide could chemically react with a thiol nucleophile such as glutathione, resulting in thiazoline ring formation (Scheme 1).^5^ In general, non-specific covalent binding of small organic molecules to proteins can lead to immune responses and induce adverse events.^7^ These findings indicate that the 2-cyanopyridine moiety in apalutamide acts as a hapten that reacts with cysteine residues in proteins, which could trigger an immune response and resulting in increased incidence of skin rash in patients.

Two reaction mechanisms for thiazoline ring formation have been proposed to date,^5,8^ one of which is described in Scheme 1.^8^ This proposed reaction mechanism suggests that cysteine-containing peptide chains could be cleaved by a reaction between 2-cyanopyridine derivatives and thiols. The nucleophilic addition of the thiol group of cysteine to 2-cyanopyridine leads to the reversible formation of thioimidate A, which is then cyclized by intramolecular nucleophilic addition of the amide nitrogen in cysteine to give 2-amidethiazolidine intermediate B. Subsequent irreversible hydrolysis of the amide bond and release of ammonia from B produces an N-terminal peptide fragment and the cyclic thiazoline-modified C-terminal fragment. By further optimizing the reaction system and simplifying the chemical structure of apalutamide, we envisioned that 2-cyanopyridine derivatives might allow cysteine-selective peptide bond cleavage.

**Scheme 2:**
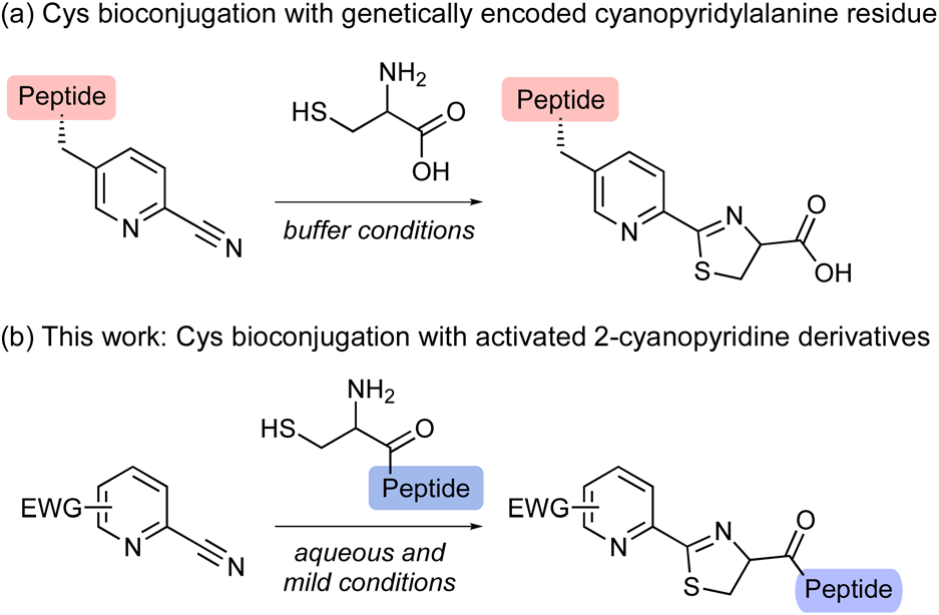
N-terminal cysteine bioconjugation by 2-cyanopyridine derivatives.

Activated heteroaromatic nitriles have recently been attracting increasing attention because of their rapid formation of a thiazoline ring with an N-terminal cysteine, as in click reactions.^9^ In particular, we were drawn to the reaction between 2-cyanopyridine derivatives and 1,2-aminothiols, which proceeds under biological conditions and can be applied to in-cell protein modifications.^10^ For example, Huber and co-workers demonstrated that the genetic encoding of 2-cyanopyridylalanine enables the site-specific attachment of a wide range of functionalities in cells (Scheme 2a).^10*a*^ However, most studies have been limited to the use of 2-cyanopyridylalanine residues, and the scope of cyanopyridine substrates that have been experimentally explored to date remains very narrow.^11^ Although electron-withdrawing substituents might increase the rate of reaction with N-terminal cysteine,^6,9*a*^ there have been no systematic investigations of the substituent effects on cyanopyridines in the reaction.

Herein, we investigated the reaction efficiency of 2-cyanopyridine derivatives bearing electron-withdrawing substituents in the reaction with cysteine (Scheme 2b). The study was inspired by the chemical reactivity of the 2-cyanopyridine moiety of apalutamide. Cysteine-selective peptide bond cleavage of glutathione was realized by using structurally optimized 2-cyanopyridine derivatives. The key features of our method are its mild reaction conditions in aqueous media and a substrate scope applicable to the bioconjugation of cysteine-containing bioactive peptides. In addition, ^15^N-labeling experiments allowed us to determine the reaction mechanism for the formation of thiazoline product from 2-cyanopyridine derivatives and cysteine.

## Results and discussion

First, we investigated the reaction between 2-cyanopyridines 1 and cysteine methyl ester 2 in an aqueous medium with THF to solubilize the substrate (Table 1). To prevent disulfide bond formation and keep Cys thiols reduced, the reactions were conducted in the presence of 4.0 equiv. of tris(2-carboxyethyl)phosphine (TCEP).^12^ As expected, the reaction of 2-cyanopyridine 1a with cysteine 2 afforded the thiazoline product 3a, in 67% yield (Table 1). It should be noted that the obtained product 3a did not show optical activity and was racemic,^13^ indicating that thiazoline products 3 racemize very rapidly.

**Table 1:** Reactions between 2-cyanopyridine derivatives and cysteine^a^

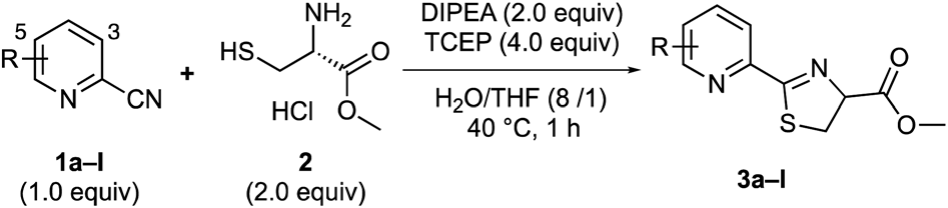			
2-Cyano pyridines	*R*	Products	Yield (%)
1a	None	3a	67
1b	3-CF_3_	3b	29
1c	5-CF_3_	3c	73
1d	3-F	3d	97
1e	5-F	3e	94
1f	5-NO_2_	3f	0
1g	5-SO_2_NH_2_	3g	53
1h	5-SO_2_NHCH_2_CH_2_Ph	3h	55
1i	5-Br	3i	79
1j	5-OMe	3j	53
1k	5-NH_2_	3k	41
1l	5-NHAc	3l	62

a Reaction conditions: 1a–l (0.3 mmol, 1.0 equiv), l-cysteine methyl ester hydrochloride 2 (2.0 equiv), DIPEA (2.0 equiv), and TCEP (4.0 equiv) in H_2_O/THF (8/1, 2.0 mL) were stirred at 40 °C for 1 h. All yields are isolated yield.

**Scheme 3:**
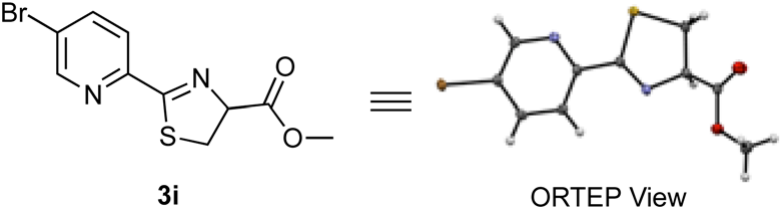
X-ray crystal structure of thiazoline 3i.

To improve the reaction efficiency, we screened several 2-cyanopyridines with various substituents on the pyridine ring (Table 1). The reaction employing 3-trifluoromethyl-2-cyanopyridine (1b), a substructure of apalutamide, decreased the reaction efficiency and the corresponding product 3b was obtained in 29% yield. Interestingly, 2-cyanopyridine 1c with a trifluoromethyl group at the 5-position slightly improved reactivity, giving the desired thiazoline 3c in 73% yield. These results indicate that the reactivity of 2-cyanopyridines with cysteine depend not only on the electronic nature of the nitrile group but also on steric hindrance around the cyanocarbon. Next, we explored the reactions with 2-cyanopyridines bearing various electron-withdrawing groups (1d–i). To our delight, the reaction with 2-cyanopyridines 1d and 1e bearing a fluoro group proceeded efficiently, affording the desired products 3d and 3e in 97% and 94% yields, respectively. In general, fluorine is highly electronegative and has a strong tendency to act as an electron-withdrawing group, which presumably contributes to the increased reactivity of the cyanocarbons. In contrast, nitro-substituted 2-cyanopyridine 1f did not provide the desired product, probably because the nitro group could be reduced to an amino group in the presence of TCEP. The reaction with 5-sulfonamide-2-cyanopyridines 1g and 1h gave the corresponding products in 53% and 55% yields, respectively. 5-Bromo-2-cyanopyridine 1i also reacted with cysteine 2 and the structure of thiazoline product 3i was confirmed to be racemic by X-ray crystal structure analysis (Scheme 3).^14^ 2-Cyanopyridines 1j–l with an electron-donating group (OMe, NH_2_, NHAc) clearly reduced reaction efficiency.

We investigated various amino acids with 2-cyanopyridine 1d to evaluate the Cys-selectivity of this ring formation reaction (see ESI†). Fortunately, 1d did not react with Ser, Thr, Lys, His, Tyr, Trp, Arg, Asp, and Glu, and the starting pyridine 1d was completely recovered. These results indicated that the developed bioconjugation reaction with 2-cyanopyridine derivatives is highly selective for cysteine.

**Fig. 2:**
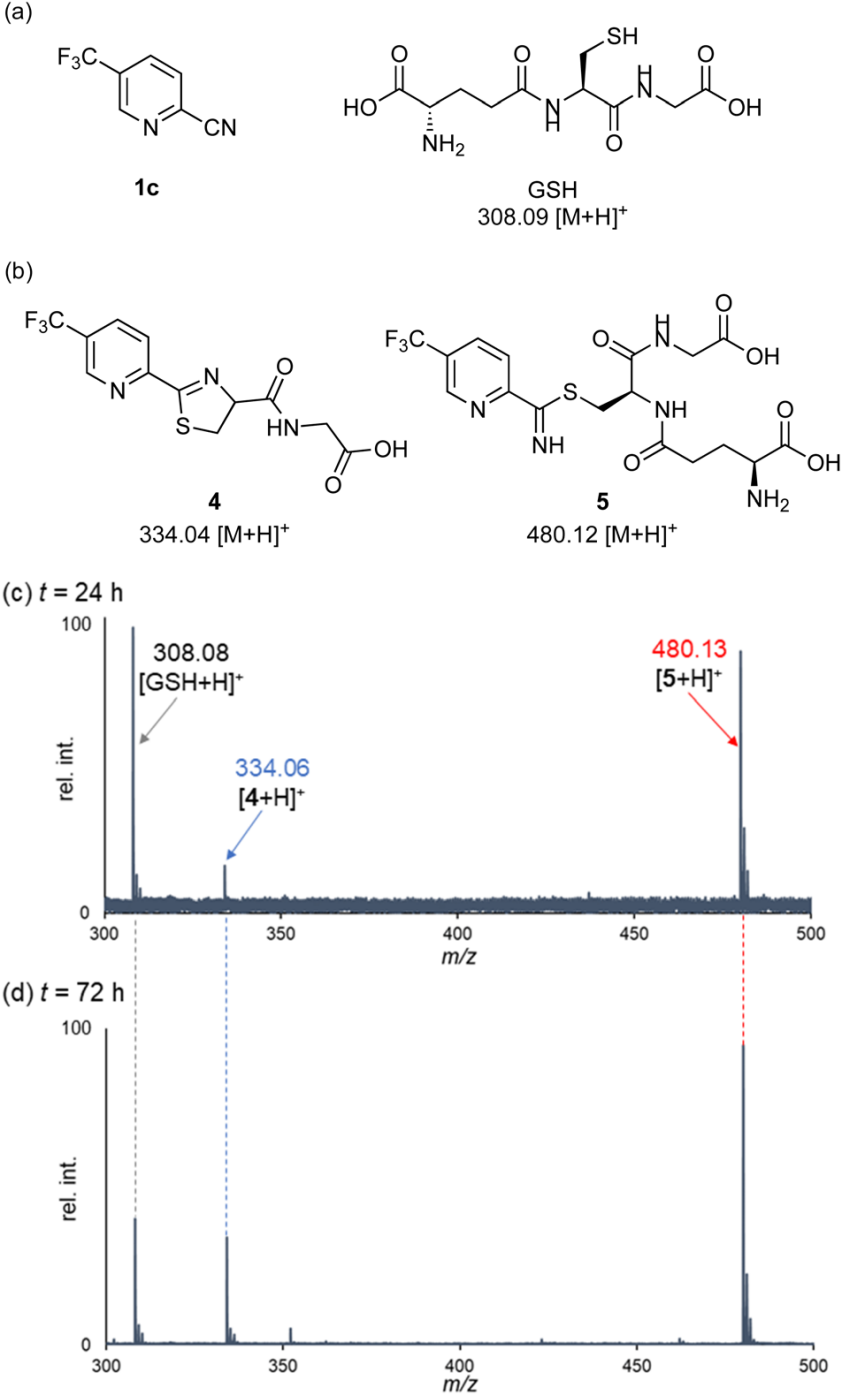
The reaction between 2-cyanopyridine 1c and glutathione. (a) Chemical structures of 1c and glutathione. (b) Plausible reaction products detected by ESI-MS. (c) ESI-MS analysis of the reaction mixture of 1c with glutathione in 50 mM ammonium acetate buffer (pH 7.0)/EtOH (4/1) at *t* = 24 h and (d) *t* = 72 h.

Having identified highly reactive 2-cyanopyridines, we next examined the peptide bond cleavage of glutathione (GSH), a tripeptide of γ-Glu-Cys-Gly (Fig. 2). Previous studies reported that apalutamide reacts with GSH under buffer conditions to form several adducts, with an elimination half-life with GSH of over 50 hours.^5^ Based on this report, we monitored the progress of the reaction between 2-cyanopyridine 1c and GSH under ammonium acetate buffer (pH 7.0) conditions using ESI-MS (Fig. 2). Stirring at 40 °C for 24 h provided two products: the thiazoline product 4 with an *m/z* of 334.06 (calcd 334.04 [M + H]^+^) and a thioimidate intermediate 5 with an *m/z* of 480.13 (calcd 480.12 [M + H]^+^). After 72 h, the concentration of GSH decreased and the relative intensity of the MS peak for the thiazoline 4 increased over time. The thioimidate 5 was formed by nucleophilic addition of the thiol group of GSH to the cyanocarbon of 1c, indicating that peptide bond cleavage proceeds *via* intramolecular cyclization from an *in situ* generated thioimidate intermediate. Monitoring the reactions between various 2-cyanopyridines with GSH showed that activated 2-cyanopyridines 1b, 1d, and 1e, bearing electron-withdrawing substituents, also cleaved the peptide bond of glutathione (see ESI†).

**Scheme 4:**
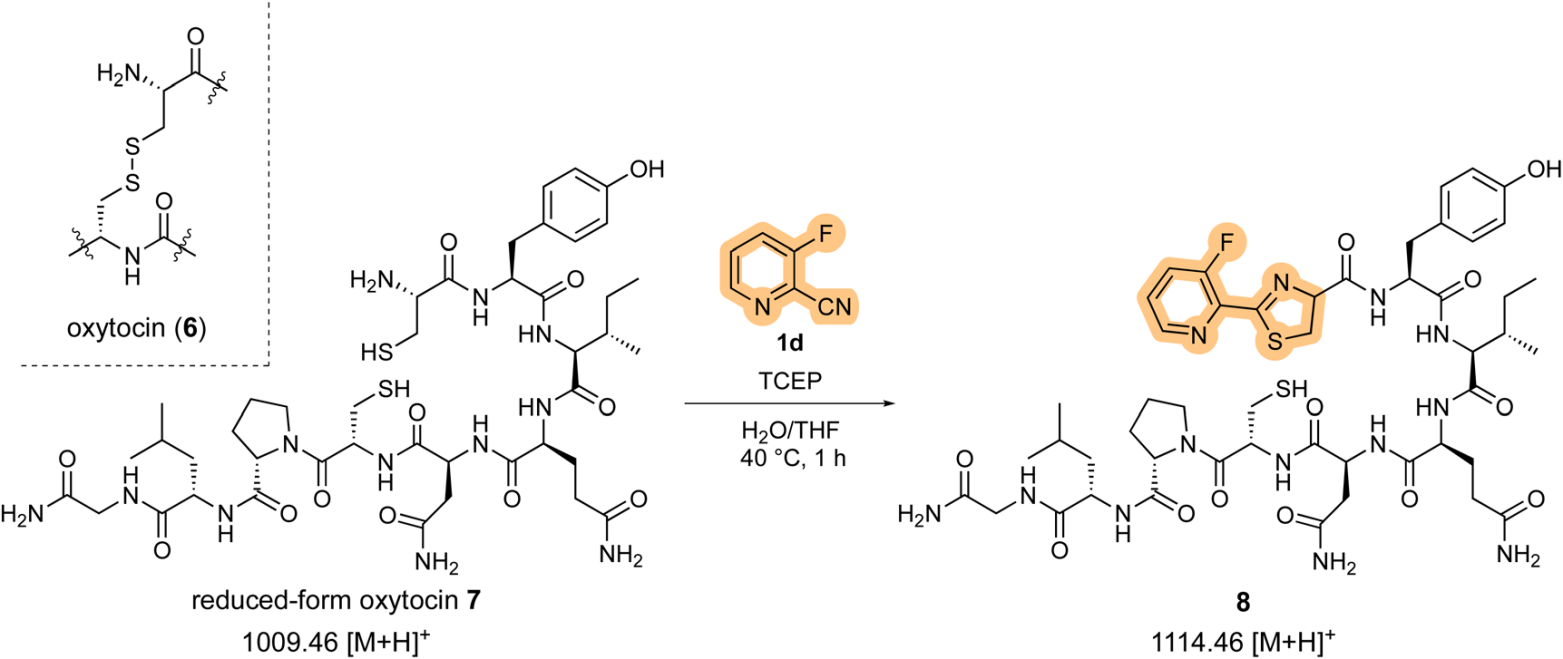
N-terminal Cys-bioconjugation of oxytocin.

To further investigate the peptide cleavage reaction and N-terminal cysteine bioconjugation, we next focused on the reaction between 2-cyanopyridine derivatives and cysteine-containing bioactive peptides. For oxytocin (6), a bioactive peptide with an intramolecular disulfide bond,^15^1d reacted only with the N-terminal cysteine residue of the *in situ* generated reduced form oxytocin 7, leaving the internal cysteine residue intact (Scheme 4).

**Fig. 3:**
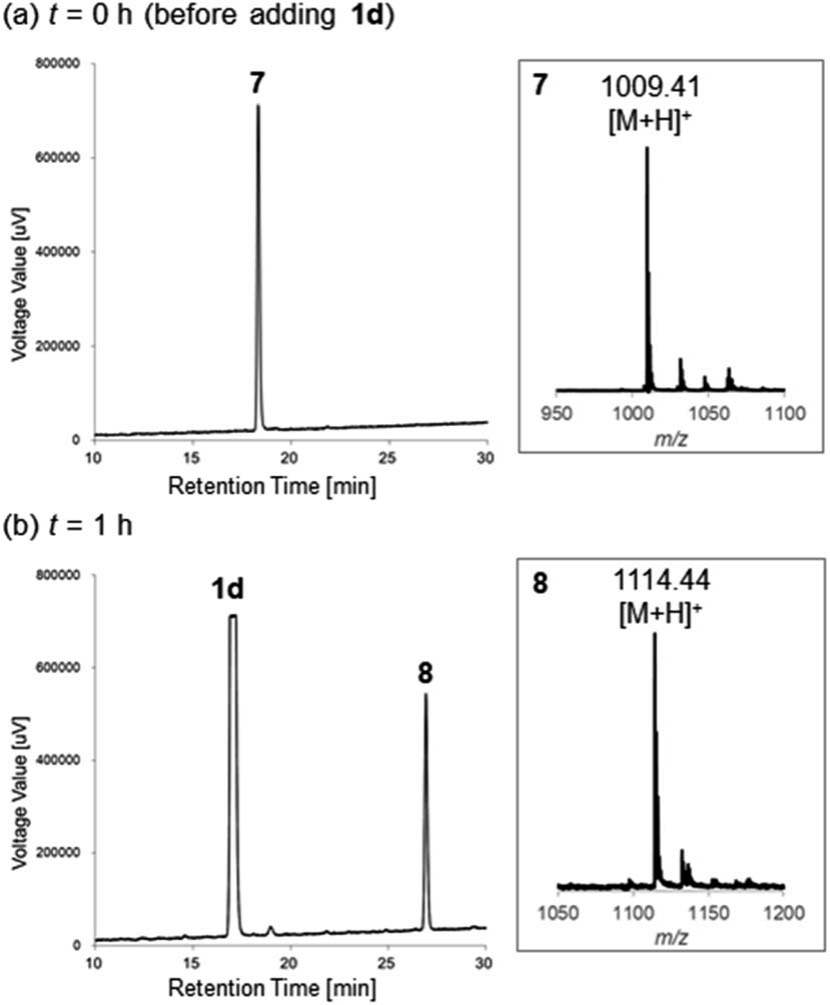
HPLC charts of the reaction between oxytocin and 1d described in Scheme 4 at (a) *t* = 0 h (before adding 1d) and (b) *t* = 1 h. The reaction was monitored by RP-HPLC (gradient: 10–40% of acetonitrile/0.1% TFA against H_2_O/0.1% TFA with a flow rate of 1.0 mL min^−1^ over 30 min). Insets show the MS corresponding peaks of reduced-form oxytocin 7 and the N-terminal thiazoline product 8.

After stirring at 40 °C for 1 h, HPLC analysis showed that the starting peptide 7 with an *m/z* = 1009.41 (calcd 1009.46 [M + H]^+^; Fig. 3a) was completely consumed and the N-terminal thiazoline product 8 with an *m/z* = 1114.44 (calcd 1114.46 [M + H]^+^; Fig. 3b) appeared as a major peak. However, cysteine-selective cleavage of the peptide bond was not observed. The same adduct formations were observed in the reaction between 1d and vasopressin (arginine vasopressin, argipressin) or lypressin (lysine vasopressin),^16^ derivatives of oxytocin bearing a similar disulfide bridge (see ESI†). These results indicate that the developed method successfully supported the N-terminal cysteine bioconjugation of bioactive oligopeptides, but cysteine-selective peptide cleavage by 2-cyanopyridine 1d may be less applicable to internal cysteine residues.

**Scheme 5:**
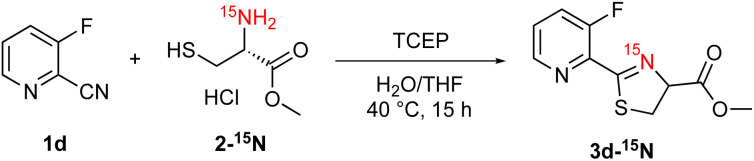
^15^N-NMR experiment of 3d-^15^N.

**Fig. 4:**
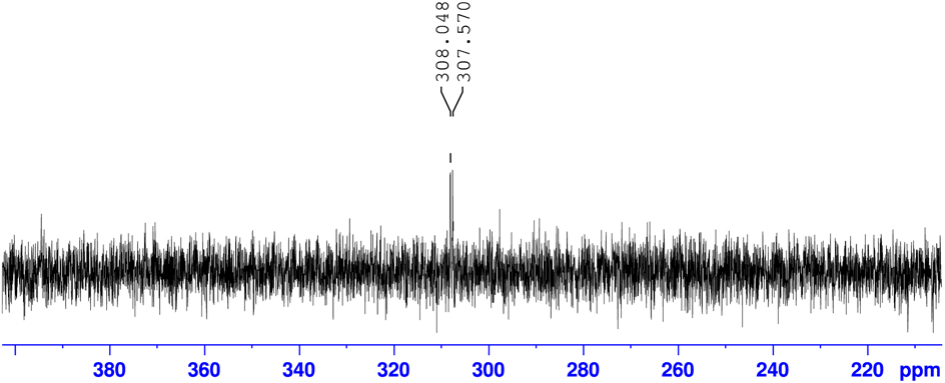
^15^N-NMR experiment of 3d-^15^N.

Additionally, we gained insight into the reaction mechanism by conducting ^15^N-labeling experiments (Scheme 5). After treating ^15^N-labeled l-cysteine methyl ester 2-^15^N with 2-cyanopyridine 1d under the optimized reaction conditions, ^15^N-NMR analysis of the obtained product revealed that the thiazoline product 3d contained ^15^N-labeled nitrogen (Fig. 4). HRMS analysis confirmed the formation of a^15^N-labeled thiazoline product,^17^ indicating that the nitrogen atom of the thiazoline ring derives from the cysteine residue. These results strongly support the reaction mechanism described in Scheme 1, including the formation of 2-amidethiazolidine intermediate B and subsequent irreversible thiazoline formation with the release of ammonia.^8,11*c*^

## Conclusion

In conclusion, we have developed an efficient method for N-terminal cysteine bioconjugation using 2-cyanopyridine derivatives. Exploration of substituent effects on the 2-cyanopyridine structure revealed that the nitrile group can be made more reactive by the installation of fluoro and trifluoromethyl groups, and the highly reactive 2-cyanopyridines allow efficient thiazoline ring formation with cysteine in aqueous media. The highlights of this reaction are its wide scope and mild reaction conditions that allow rapid bioconjugation with bioactive peptides. Moreover, it is noteworthy that the highly reactive 2-cyanopyridines enable the peptide bond cleavage of glutathione. The developed peptide cleavage reaction using 2-cyanopyridine derivatives holds promise as a valuable chemical tool for site-selective peptide modifications. Further applications to realize the cysteine-selective bond cleavage of oligopeptides and protein chains are currently underway in our laboratory.

## Conflicts of interest

There are no conflicts to declare.

## Supplementary Material

RA-014-D4RA00437J-s001

RA-014-D4RA00437J-s002
