# Correction: 2-cyanopyridine derivatives enable N-terminal cysteine bioconjugation and peptide bond cleavage of glutathione under aqueous and mild conditions

**DOI:** 10.1039/d4ra90039a

**Published:** 2024-04-17

**Authors:** Tetsuya Yano, Takahiro Yamada, Hiroaki Ishida, Nami Ohashi, Toshimasa Itoh

**Affiliations:** a Showa Pharmaceutical University Machida Tokyo 194-8543 Japan t-yamada@ac.shoyaku.ac.jp titoh@ac.shoyaku.ac.jp

## Abstract

Correction for ‘2-cyanopyridine derivatives enable N-terminal cysteine bioconjugation and peptide bond cleavage of glutathione under aqueous and mild conditions’ by Tetsuya Yano *et al.*, *RSC Adv.*, 2024, **14**, 6542–6547, https://doi.org/10.1039/D4RA00437J.

The authors regret that the name of one of the authors (Hiroaki Ishida) was shown incorrectly in the original article. The corrected author list is as shown above.

The Royal Society of Chemistry apologises for these errors and any consequent inconvenience to authors and readers.

## Supplementary Material

