# Supplementary material for: 2-cyanopyridine derivatives enable N-terminal cysteine bioconjugation and peptide bond cleavage of glutathione under aqueous and mild conditions
Source: RSC Adv. 2024 Feb 22;14(10):6542–7. doi: 10.1039/d4ra00437j (PMC10882492; doi:10.1039/d4ra00437j)
Supplement: RA-014-D4RA00437J-s001 [file RA-014-D4RA00437J-s001.pdf]

Electronic Supplementary Material (ESI) for RSC Advances.
This journal is © The Royal Society of Chemistry 2024

2-Cyanopyridine derivatives enable N-terminal cysteine bioconjugation and peptide bond cleavage of glutathione under aqueous and mild conditions

Supporting Information

Tetsuya Yano, Takahiro Yamada*, Hiroaki Ishida, Nami Ohashi and Toshimasa Itoh*

Showa Pharmaceutical University, 3-3165, Higashi-Tamagawagakuen, Machida, Tokyo 194-8543, Japan

E-mail: t-yamada@ac.shoyaku.ac.jp; titoh@ac.shoyaku.ac.jp

Table of contents

General methods S2
Preparation of 2-cyanopyridine derivatives S2
General procedure for the reaction between 2-cyanopyridines and cysteine S4
The reaction between 2-cyanopyridine derivatives and cysteine S4
The reaction between various amino acids and 2-cyanopyridine **1d** S10
Peptide bond cleavage of glutathione with activated 2-cyanopyridine derivatives S11
N-Terminal cysteine-bioconjugation of bioactive peptides S14
^{15}N-Labeling experiments S17
Single-crystal X-ray diffraction analysis S19
CD spectra of thiazoline products S21
References S22
NMR data of new compounds S23

General methods

^{1}H and ^{13}C NMR spectra were recorded with BRUKER AV300M and BRUKER AV400NEO spectrometers at room temperature, with tetramethylsilane (δ = 0) as an internal standard ($CDCl_3$ or MeOH-d_4). Chemical shifts were expressed in ppm, and coupling constants (*J*) in Hz. High-resolution mass spectrometry (HRMS) data were recorded on JEOL JMS-700 and JMS-T100LP spectrometers. Melting points were determined by using a Yanaco melting point apparatus MP-S3. Crystal data were collected using a Rigaku XtalLAB Synergy Custom (Custom-made machine). Reverse-phase HPLC (RP-HPLC) was performed with a JASCO Gulliver system equipped with an Intelligent HPLC Pump PU-980 and an Intelligent UV/VIS Detector UV-970, and the eluent was detected by UV at 220 or 254 nm. For analytical RP-HPLC, a COSMOSIL 5C18-AR-II (4.6 × 250 mm) packed column was employed with linear gradients of acetonitrile and H_2O containing 0.1% (v/v) TFA with a flow rate of 1.0 mL min^{-1}. For preparative RP-HPLC, a Shim-pack PREP-ODS (20 × 250 mm) column was employed with linear gradients of acetonitrile and H_2O containing 0.1% (v/v) TFA with a flow rate of 10 mL min^{-1}. CD spectra were recorded on a Jasco J-1500 Circular Dichroism spectrometer at 25 °C. The CD spectra were measured at 0.1 nm spectral resolution using a 1 mm path length quartz cuvette and the scan rate of each spectrum was 100 nm/min. Wako silica gel 70 F254 and Fuji Silysia CHROMATOREX NH-TLC were used for thin layer chromatography (TLC). Kanto Chemical silica gel 60N (spherical neutral 40–50 μm) and Fuji Silysia CHROMATOREX NH-DM1020 (100 μm) were used for column chromatography.

Preparation of 2-cyanopyridine derivatives

2-Cyanopyridines **1a**, **1b**, **1c**, **1d**, **1e**, **1f**, **1i**, **1j**, and **1k** were purchased from commercial sources and used without further purification.

2-Cyanopyridines **1g** and **1l** were synthesized according to the following procedures.[1,2]

2-Cyanopyridine **1h** was synthesized according to the following procedure.

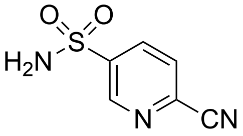

1g

6-Cyanopyridine-3-sulfonamide (**1g**)[1]: White solid. mp 142–143 °C. ^{1}H NMR (300 MHz, MeOH-*d*$_4$) δ 9.15 (dd, *J* = 2.2, 0.8 Hz, 1H, Ar-H), 8.42 (dd, *J* = 8.2, 2.2 Hz, 1H, Ar-H), 8.06 (dd, *J* = 8.2, 0.8 Hz, 1H, Ar-H). ^{13}C NMR (75 MHz, MeOH-*d*$_4$) δ 148.1, 143.0, 135.6, 135.3, 128.7, 116.1.

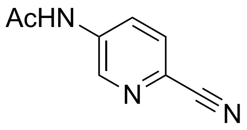

1l

N-(6-Cyanopyridin-3-yl)acetamide (**1l**)[2]: White solid. mp 177–178 °C. ^{1}H NMR (400 MHz, DMSO-*d*$_6$) δ 10.61 (s, 1H, NH), 8.81 (d, *J* = 2.0 Hz, 1H, Ar-H), 8.24 (dd, *J* = 8.6, 2.0 Hz, 1H, Ar-H), 7.94 (d, *J* = 8.6 Hz, 1H, Ar-H), 2.12 (s, 3H, Ac). ^{13}C NMR (100 MHz, DMSO-*d*$_6$) δ 170.1, 142.1, 139.6, 130.1, 126.2, 125.9, 118.2, 24.5. HRMS (ESI) *m/z* calcd for $C_8H_7N_3O+Na^+$: 184.0487 $[M+Na]^+$; found: 184.0490.

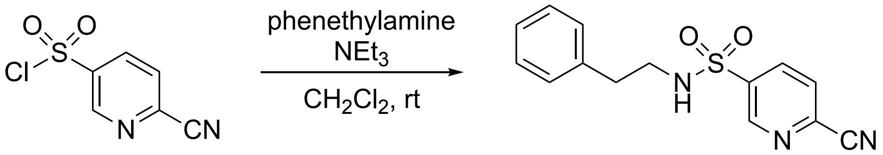

1h

6-Cyano-*N*-phenethylpyridine-3-sulfonamide (**1h**): To a stirred solution of 6-cyanopyridine-3-sulfonyl chloride (380 mg, 1.88 mmol, 1.1 equiv.) in CH_2Cl_2 (2 mL) were added phenethylamine (0.22 mL, 1.71 mmol, 1.0 equiv.) and triethylamine (0.36 mL, 1.5 equiv.) at 0 °C. After stirring at room temperature for 2 h, the reaction mixture was concentrated in *vacuo*. The crude residue was purified by flash chromatography on silica gel (hexane : AcOEt = 1:1) to afford the corresponding product **1h** (277 mg, 56% yield) as a colorless oil. ^{1}H NMR (300 MHz, $CDCl_3$) δ 9.01 (dd, *J* = 2.2, 0.8 Hz, 1H, Py-H), 8.12 (dd, *J* = 8.1, 2.2 Hz, 1H, Py-H), 7.76 (dd, *J* = 8.1, 0.8 Hz, 1H, Py-H), 7.28–7.17 (m, 3H, Ph-H), 7.12–7.05 (m, 2H, Ph-H), 5.14 (t, *J* = 6.0 Hz, 1H, NH), 3.33 (td, *J* = 6.8, 6.0 Hz, 2H, CH_2), 2.81 (t, *J* = 6.8 Hz, 2H, CH_2). ^{13}C NMR (75 MHz, $CDCl_3$) δ 148.9, 139.6, 136.9, 136.5, 135.6, 128.9, 128.7, 128.4, 127.1, 116.0, 44.4, 35.8. HRMS (ESI) *m/z* calcd for $C_{14}H_{13}N_3O_2S+Na^+$: 310.0626 $[M+Na]^+$; found: 310.0627.

General procedure for the reaction between 2-cyanopyridines and cysteine

General procedure: To a solution of 2-cyanopyridine **1** (0.3 mmol, 1.0 equiv.), *L*-cysteine methyl ester hydrochloride **2** (0.6 mmol, 2.0 equiv.) in THF (0.3 mL) and 0.5 M TCEP (pH = 7.0) aqueous solution (2.4 mL, 4.0 equiv.) was added DIPEA (105 μL, 2.0 equiv.). After stirring at 40 °C (silicone oil bath) for 1 h, the reaction was quenched with saturated $NaHCO_3$ aq. The aqueous layer was extracted with AcOEt and the combined organic layers were washed with brine, dried over Na_2SO_4, filtered and concentrated in *vacuo*. The crude product was purified by flash column chromatography on silica gel to afford the corresponding products **3**. The obtained thiazolines **3a**, **3d** and **3i** did not show optical activity and the single-crystal X-ray diffraction analysis of thiazoline **3i** confirmed that the thiazoline products were obtained as a racemic form. In the case of 2-cyanopyridine **1a**, a trace amount of thioimidate intermediate **3a'** was isolated.

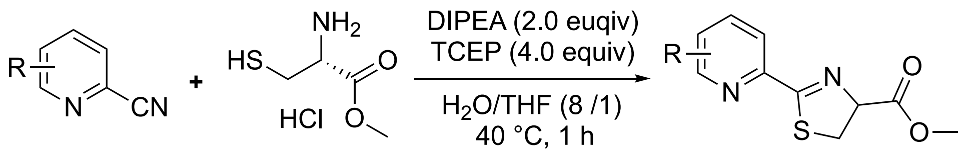

1a–l (1.0 equiv) + **2** (2.0 equiv) → **3a–l**

The reaction between 2-cyanopyridine derivatives and cysteine

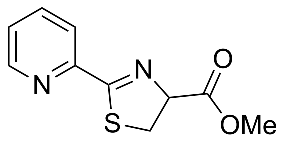

3a

Following the general procedure with 2-cyanopyridine **1a** (31 mg, 0.3 mmol), purification by flash column chromatography (hexane : AcOEt = 2 : 1) afforded the corresponding product **3a** (45 mg, 67% yield) as a colorless oil and a trace amount of thioimidate **3a'** as a colorless oil.

Methyl 2-(pyridin-2-yl)-4,5-dihydrothiazole-4-carboxylate (**3a**): Colorless oil. ^{1}H NMR (400 MHz, $CDCl_3$) δ 8.70–8.65 (m, 1H, Ar-H), 8.16 (ddd, *J* = 7.9, 2.2, 1.2 Hz, 1H, Ar-H), 7.78 (ddd, *J* = 7.9, 7.7, 1.2 Hz, 1H, Ar-H), 7.78 (ddd, *J* = 7.9, 6.2, 1.2 Hz, 1H, Ar-H), 5.38 (t, *J* = 9.5 Hz, 1H, CH), 3.85 (s, 3H, Me), 3.71–3.55 (m, 2H, CH_2).^{13}C NMR (100 MHz, $CDCl_3$) δ 173.5, 171.2, 150.5, 149.3, 136.6, 125.8, 121.9, 78.9, 52.8, 34.3. HRMS (ESI) *m/z* calcd for $C_{10}H_{10}N_2O_2S+Na^+$: 245.0361 $[M+Na]^+$; found: 245.0363.

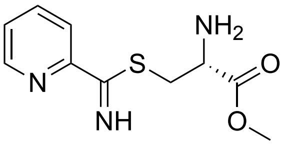
3a'

3a': Colorless oil. ^{1}H NMR (400 MHz, $CDCl_3$) δ 10.88 (br, 1H, NH), 8.67 (dd, *J* = 8.0, 1.0 Hz, 1H, Ar-H), 8.60 (ddd, *J* = 8.0, 1.7, 1.0 Hz, 1H, Ar-H), 7.87 (ddd, *J* = 8.0, 7.7, 1.7 Hz, 1H, Ar-H), 7.49 (ddd, *J* = 8.0, 7.7, 1.0 Hz, 1H, Ar-H), 5.68–5.60 (m, 1H, CH), 3.47 (s, 3H, Me), 3.47–3.38 (m, 1H, CH_2), 3.25–3.10 (m, 1H, CH_2). ^{13}C NMR (100 MHz, $CDCl_3$) δ 191.6, 169.8, 150.7, 147.3, 137.2, 126.4, 124.8, 58.9, 53.0, 25.6.

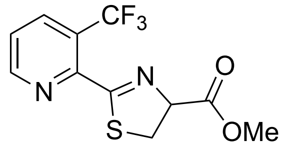
3b

Following the general procedure with 3-trifluoromethyl-2-cyanopyridine **1b** (52 mg, 0.3 mmol), purification by flash column chromatography (hexane : AcOEt = 2 : 1) afforded the corresponding product **3b** (25 mg, 29% yield) as a colorless oil.

Methyl 2-(3-(trifluoromethyl)pyridin-2-yl)-4,5-dihydrothiazole-4-carboxylate (**3b**): Colorless oil. ^{1}H NMR (300 MHz, $CDCl_3$) δ 8.81 (d, *J* = 4.5 Hz, 1H, Ar-H), 8.12 (d, *J* = 8.1 Hz, 1H, Ar-H), 7.53 (dd, *J* = 8.1, 4.5 Hz, 1H, Ar-H), 5.44 (t, *J* = 9.4 Hz, 1H, CH), 3.84 (s, 3H, Me), 3.84–3.60 (m, 2H, CH_2). ^{13}C NMR (75 MHz, $CDCl_3$) δ 170.5, 169.0, 151.5, 149.7, 135.4 (q, *J* = 5.2 Hz), 125.2 (q, *J* = 33.6 Hz), 124.5, 122.6 (q, *J* = 272 Hz), 79.7, 52.8, 35.2. HRMS (ESI) *m/z* calcd for $C_{11}H_9F_3N_2O_2S+Na^+$: 313.0235 $[M+Na]^+$; found: 313.0232.

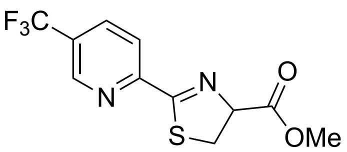
3c

Following the general procedure with 5-trifluoromethyl-2-cyanopyridine **1c** (52 mg, 0.3 mmol), purification by flash column chromatography (hexane : AcOEt = 4 : 1) afforded the corresponding product **3c** (64 mg, 73% yield) as a pale yellow solid.

Methyl 2-(5-(trifluoromethyl)pyridin-2-yl)-4,5-dihydrothiazole-4-carboxylate (**3c**): Pale yellow solid. mp 67–68 °C. ^{1}H NMR (400 MHz, $CDCl_3$) δ 8.91 (d, *J* = 2.2 Hz, 1H, Ar-H), 8.29 (d, *J* = 8.3 Hz, 1H, Ar-H), 8.03 (dd, *J* = 8.3, 2.2 Hz, 1H, Ar-H), 5.40 (t, *J* = 9.6 Hz, 1H, CH), 3.86 (s, 3H, Me), 3.76–3.60 (m, 2H, CH_2). ^{13}C NMR (75 MHz, $CDCl_3$) δ 172.4, 170.8, 153.4, 146.2, 133.8, 128.2 (q, *J* = 33.2 Hz), 123.1 (q, *J* = 271 Hz), 121.6, 78.9, 52.9, 34.4. HRMS (ESI) *m/z* calcd for $C_{11}H_9F_3N_2O_2S+Na^+$: 313.0235 $[M+Na]^+$; found: 313.0224.

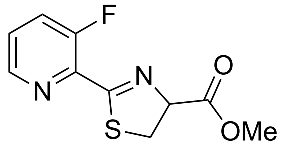

3d

Following the general procedure with 3-fluoro-2-cyanopyridine **1d** (37 mg, 0.3 mmol), purification by flash column chromatography (hexane : AcOEt = 2 : 1) afforded the corresponding product **3d** (70 mg, 97% yield) as a white solid. When 0.5 M TCEP (pH = 4.0 or pH = 9.0) was used instead of TCEP (pH = 7.0) aqueous solution, the product yield decreased to 68% or 30%, respectively.

Methyl 2-(3-fluoropyridin-2-yl)-4,5-dihydrothiazole-4-carboxylate (**3d**): White solid. mp 82–83 °C. ^{1}H NMR (300 MHz, $CDCl_3$) δ 8.54–8.49 (m, 1H, Ar-H), 7.56 (dd, *J* = 10.0, 8.4 Hz, 1H, Ar-H), 7.49–7.41 (m, 1H, Ar-H), 5.49 (t, *J* = 9.3 Hz, 1H, CH), 3.84 (s, 3H, Me), 3.66–3.53 (m, 2H, CH_2). ^{13}C NMR (75 MHz, $CDCl_3$) δ 171.0, 169.6 (d, *J* = 9.3 Hz), 157.5 (d, *J* = 270 Hz), 144.9 (d, *J* = 5.1 Hz), 138.6 (d, *J* = 8.2 Hz), 127.0 (d, *J* = 4.8 Hz), 125.0 (d, *J* = 19.3 Hz), 80.0, 52.8, 33.4. HRMS (ESI) *m/z* calcd for $C_{10}H_9FN_2O_2S+Na^+$: 263.0266 $[M+Na]^+$; found: 263.0264.

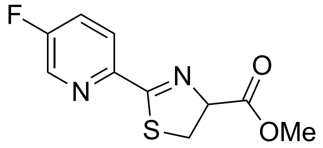

3e

Following the general procedure with 5-fluoro-2-cyanopyridine **1e** (37 mg, 0.3 mmol), purification by flash column chromatography (hexane : AcOEt = 2 : 1) afforded the corresponding product **3e** (68 mg, 94% yield) as a pale yellow solid.

Methyl 2-(5-fluoropyridin-2-yl)-4,5-dihydrothiazole-4-carboxylate (**3e**): Pale yellow solid. mp 53–54 °C. ^{1}H NMR (300 MHz, $CDCl_3$) δ 8.46 (d, *J* = 2.8 Hz, 1H, Ar-H), 8.16 (dd, *J* = 8.8, 4.5 Hz, 1H, Ar-H), 7.46 (ddd, *J* = 8.8, 4.5, 2.8 Hz, 1H, Ar-H), 5.33 (t, *J* = 9.4 Hz, 1H, CH), 3.81 (s, 3H, Me), 3.68–3.50 (m, 2H, CH_2). ^{13}C NMR (100 MHz, $CDCl_3$) δ 172.1, 171.1, 161.9, 159.3, 146.9, 137.6 (d, *J* = 24.6 Hz), 123.4, 78.9, 52.8, 34.6. HRMS (ESI) *m/z* calcd for $C_{10}H_9FN_2O_2S+Na^+$: 263.0267 $[M+Na]^+$; found: 263.0273.

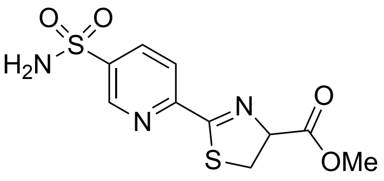

3g

Following the general procedure with 6-cyanopyridine-3-sulfonamide **1g** (55 mg, 0.3 mmol), purification by flash column chromatography (hexane : AcOEt = 1 : 2) afforded the corresponding product **3g** (48 mg, 53% yield) as a white solid.

Methyl 2-(5-sulfamoylpyridin-2-yl)-4,5-dihydrothiazole-4-carboxylate (**3g**): White solid. mp 163–164 °C. ^{1}H NMR (400 MHz, DMSO-*d*$_6$) δ 9.04 (dd, *J* = 2.2, 0.9 Hz, 1H, Ar-H), 8.33 (dd, *J* = 8.3, 2.2 Hz, 1H, Ar-H), 8.26 (dd, *J* = 8.3, 0.9 Hz, 1H, Ar-H), 5.56 (t, *J* = 9.3 Hz, 1H, CH), 3.82 (s, 3H, Me), 3.75–3.55 (m, 2H, CH_2).^{13}C NMR (100 MHz, DMSO-*d*$_6$)) δ 171.5, 171.1, 152.4, 146.8, 142.4, 135.5, 122.2, 78.9, 53.0, 34.3. HRMS (ESI) *m/z* calcd for $C_{10}H_{11}N_3O_4S_2$+Na^+: 324.0089 [M+Na]$^+$; found: 324.0087.

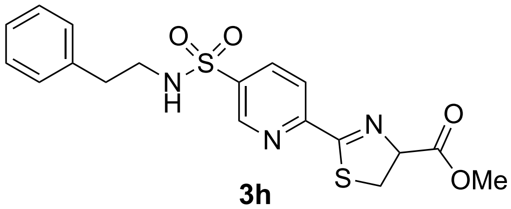

3h

Following the general procedure with 2-cyanopyridine derivative **1h** (29 mg, 0.1 mmol), purification by flash column chromatography (hexane : AcOEt = 2 : 1) afforded the corresponding product **3h** (23 mg, 55% yield) as a pale yellow oil.

Methyl 2-(5-(*N*-phenethylsulfamoyl)pyridin-2-yl)-4,5-dihydrothiazole-4-carboxylate (**3h**): Pale yellow oil. ^{1}H NMR (400 MHz, $CDCl_3$) δ 9.01 (d, *J* = 2.1 Hz, 1H, Py-H), 8.24 (d, *J* = 8.3 Hz, 1H, Py-H), 8.10 (dd, *J* = 8.3, 2.1 Hz, 1H, Py-H), 7.29–7.20 (m, 3H, Ph-H), 7.09 (d, *J* = 7.6 Hz, 2H, Ph-H), 5.42 (t, *J* = 9.5 Hz, 1H, CH), 4.80–4.70 (m, 1 H, NH), 3.88 (s, 3H, Me), 3.85–3.64 (m, 2H, CH_2), 3.40–3.20 (m, 2H, CH_2), 2.81 (t, *J* = 6.8 Hz, 2H, CH_2).^{13}C NMR (100 MHz, $CDCl_3$) δ 172.2, 170.8, 153.3, 147.4, 138.2, 137.1, 135.2, 128.9, 128.6, 127.0, 121.9, 79.0, 52.9, 44.2, 35.8, 34.5. HRMS (ESI) *m/z* calcd for $C_{18}H_{19}N_3O_4S_2$+Na^+: 428.0715 [M+Na]$^+$; found: 428.0706.

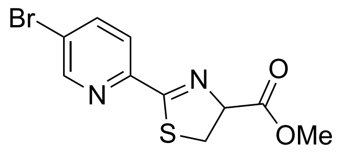

3i

Following the general procedure with 5-bromo-2-cyanopyridine **1i** (50 mg, 0.3 mmol), purification by flash column chromatography (hexane : AcOEt = 3 : 1) afforded the corresponding product **3i** (64 mg, 79% yield) as a pale yellow solid.

Methyl 2-(5-bromopyridin-2-yl)-4,5-dihydrothiazole-4-carboxylate (**3i**): Pale yellow solid. mp 75–76 °C. ^{1}H NMR (400 MHz, $CDCl_3$) δ 8.73 (dd, J = 2.4, 0.8 Hz, 1H, Ar-H), 8.07 (dd, J = 8.4, 0.8 Hz, 1H, Ar-H), 7.94 (dd, J = 8.4, 2.4 Hz, 1H, Ar-H), 5.37 (t, J = 9.6 Hz, 1H, CH), 3.87 (s, 3H, Me), 3.75–3.60 (m, 2H, CH_2).^{13}C NMR (100 MHz, $CDCl_3$) δ 172.5, 171.0, 150.4, 149.0, 139.2, 123.5, 123.0, 79.0, 52.9, 34.5. HRMS (ESI) *m/z* calcd for $C_{10}H_9BrN_2O_2S$+Na^+: 322.9466 $[M+Na]^+$; found: 322.9454.

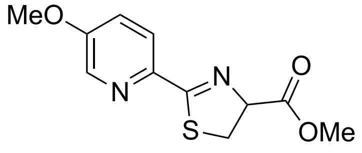

3j

Following the general procedure with 5-methoxy-2-cyanopyridine **1j** (40 mg, 0.3 mmol), purification by flash column chromatography (hexane : AcOEt = 1 : 1) afforded the corresponding product **3j** (40 mg, 53% yield) as a white solid.

Methyl 2-(5-methoxypyridin-2-yl)-4,5-dihydrothiazole-4-carboxylate (**3j**): White solid. mp 72–73 °C. ^{1}H NMR (400 MHz, $CDCl_3$) δ 8.32 (d, J = 3.2 Hz, 1H, Ar-H), 8.11 (d, J = 8.8 Hz, 1H, Ar-H), 7.25 (dd, J = 8.8, 3.2 Hz, 1H, Ar-H), 5.34 (t, J = 9.4 Hz, 1H, CH), 3.91 (s, 3H, Me), 3.84 (s, 3H, Me), 3.68–3.56 (m, 2H, CH_2). ^{13}C NMR (100 MHz, $CDCl_3$) δ 172.6, 171.4, 157.4, 143.2, 137.0, 122.8, 120.2, 78.8, 55.7, 52.7, 34.3. HRMS (ESI) *m/z* calcd for $C_{11}H_{12}N_2O_3S$+Na^+: 275.0466 $[M+Na]^+$; found: 275.0454.

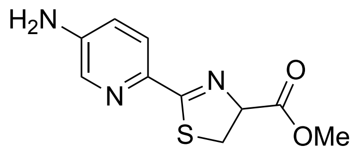

3k

Following the general procedure with 5-amino-2-cyanopyridine **1k** (36 mg, 0.3 mmol), purification by flash column chromatography (hexane : AcOEt = 1 : 4) afforded the corresponding product **3k** (29 mg, 41% yield) as a pale yellow solid.

Methyl 2-(5-aminopyridin-2-yl)-4,5-dihydrothiazole-4-carboxylate (**3k**): Pale yellow solid. mp 139–140 °C. ^{1}H NMR (400 MHz, $CDCl_3$) δ 8.06 (dd, *J* = 2.4, 0.4 Hz, 1H, Ar-H), 7.95 (d, *J* = 8.4 Hz, 1H, Ar-H), 6.97 (dd, *J* = 8.4, 2.4 Hz, 1H, Ar-H), 5.32 (t, *J* = 9.2 Hz, 1H, CH), 4.12 (br, 2H, NH_2), 3.84 (s, 3H, Me), 3.65–3.50 (m, 2H, CH_2).^{13}C NMR (100 MHz, $CDCl_3$) δ 172.9, 171.6, 144.8, 140.8, 136.0, 122.9, 120.6, 78.7, 52.7, 34.2. HRMS (ESI) *m/z* calcd for $C_{10}H_{11}N_3O_2S_2$+Na^+: 260.0470 $[M+Na]^+$; found: 260.0470.

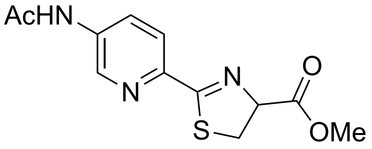

3l

Following the general procedure with *N*-(6-cyanopyridin-3-yl)acetamide **1l** (48 mg, 0.3 mmol), purification by flash column chromatography (AcOEt only) afforded the corresponding product **3l** (52 mg, 62% yield) as a white solid.

Methyl 2-(5-acetamidopyridin-2-yl)-4,5-dihydrothiazole-4-carboxylate (**3l**): White solid. mp 176–177 °C. ^{1}H NMR (400 MHz, $CDCl_3$) δ 8.59 (d, *J* = 2.4 Hz, 1H, Ar-H), 8.26 (dd, *J* = 8.4, 2.0 Hz, 1H, Ar-H), 8.08 (d, *J* = 8.4 Hz, 1H, Ar-H), 8.00 (br, 1H, NH), 5.38 (t, *J* = 9.2 Hz, 1H, CH), 3.87 (s, 3H, Me), 3.70–3.55 (m, 2H, CH_2), 2.23 (s, 3H, Ac). ^{13}C NMR (100 MHz, $CDCl_3$) δ 172.7, 171.5, 168.9, 145.7, 139.8, 139.7, 136.9, 136.8, 126.5, 126.4, 122.5, 78.8, 52.8, 34.4, 24.5. HRMS (ESI) *m/z* calcd for $C_{12}H_{13}N_3O_3S$+Na^+: 302.0575 $[M+Na]^+$; found: 302.0564.

In the case of 2-cyano-5-nitropyridine **1f** , the reaction resulted a messy mixture and the desired product **3f** could not be detected. When the reaction with 3-nitro-2-cyanopyridine, the reaction also resulted a messy mixture but a trace amount of the reduction product was obtained as described below. Its structure was confirmed by ^{1}H NMR and X-ray crystal structure analysis. This result suggests that the nitro group could be reduced to amino group under the reaction conditions, which is probably the main decomposition pathway of **1f**.

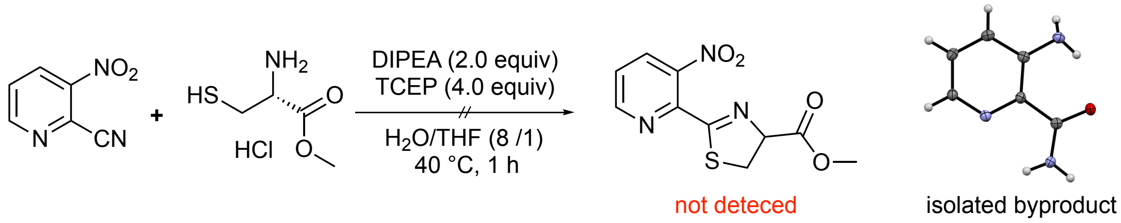

The reaction between various amino acids and 2-cyanopyridine 1d

To a solution of 2-cyanopyridine **1d** (0.3 mmol, 1.0 equiv.) and amino acids (0.6 mmol, 2.0 equiv.) in THF (0.3 mL) and H_2O (2.4 mL) was added DIPEA (2.0–4.0 equiv.). After stirring at 40 °C (silicone oil bath) for 1 h, the reaction was quenched with saturated NH_4Cl aq. The aqueous layer was extracted with AcOEt and the combined organic layers were washed with brine, dried over Na_2SO_4, filtered and concentrated in *vacuo*. The crude product was purified by flash column chromatography on silica gel.

Table S1. Screening of the reaction between various amino acids and **1d**.

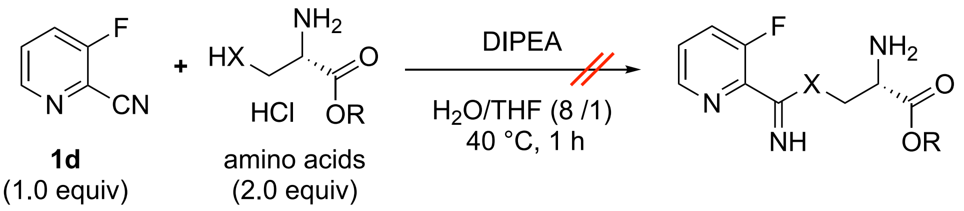

Entry	Amino acids (2.0 equiv)	DIPEA	Results	Recovery of **1d** (%)
1	H_2N-Ser-OMe · HCl	2.0 equiv	no reaction	>99
2	H_2N-Thr-OBn · HCl	2.0 equiv	no reaction	97
3	H_2N-Lys-OMe · 2HCl	4.0 equiv	no reaction	>99
4	H_2N-His-OMe · 2HCl	4.0 equiv	no reaction	>99
5	H_2N-Tyr-OMe · HCl	2.0 equiv	no reaction	81
6	H_2N-Trp-OMe · HCl	2.0 equiv	no reaction	86
7	H_2N-Arg-OMe · 2HCl	4.0 equiv	no reaction	92
8	H_2N-Asp-OMe	none[a]	no reaction	>99
9	H_2N-Glu-OMe	none[a]	no reaction	>99

a) The reactions were conducted without DIPEA.

Peptide bond cleavage of glutathione with activated 2-cyano pyridine derivatives

Representative procedure for the reaction between 2-cyanopyridines and glutathione

To a solution of 5-trifluoromethyl-2-cyanopyridine (**1c**, 50 mg, 0.29 mmol, 48 mM) in EtOH (1.2 mL) was added a solution of glutathione (356 mg, 4.0 equiv., 190 mM) in 50 mM ammonium acetate buffer (pH 7.0, 4.8 mL). The reaction mixture was stirred at 40 °C (silicone oil bath) and progress of the reaction was monitored over time by ESI-MS analysis in positive detection mode. The use of ammonium acetate buffer (pH 7.0) solution allowed direct injection of the reaction mixture.

The reaction between 2-cyanopyridine 1b and glutathione

Following the representative procedure described above, the progress of the reaction between 2-cyanopyridine **1b** and glutathione was monitored by ESI-MS analysis at 48 h and 72 h.

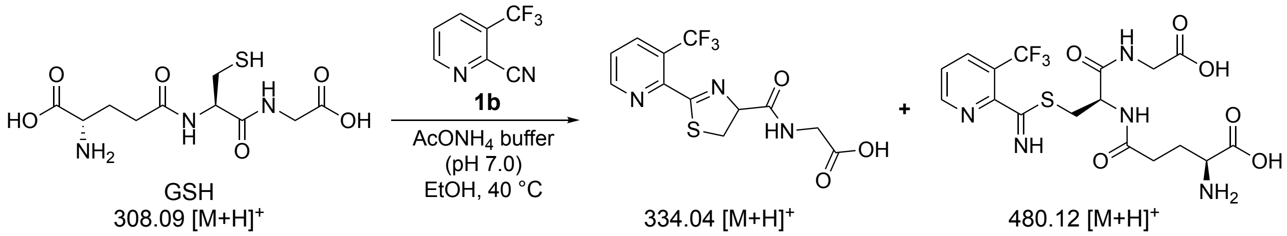

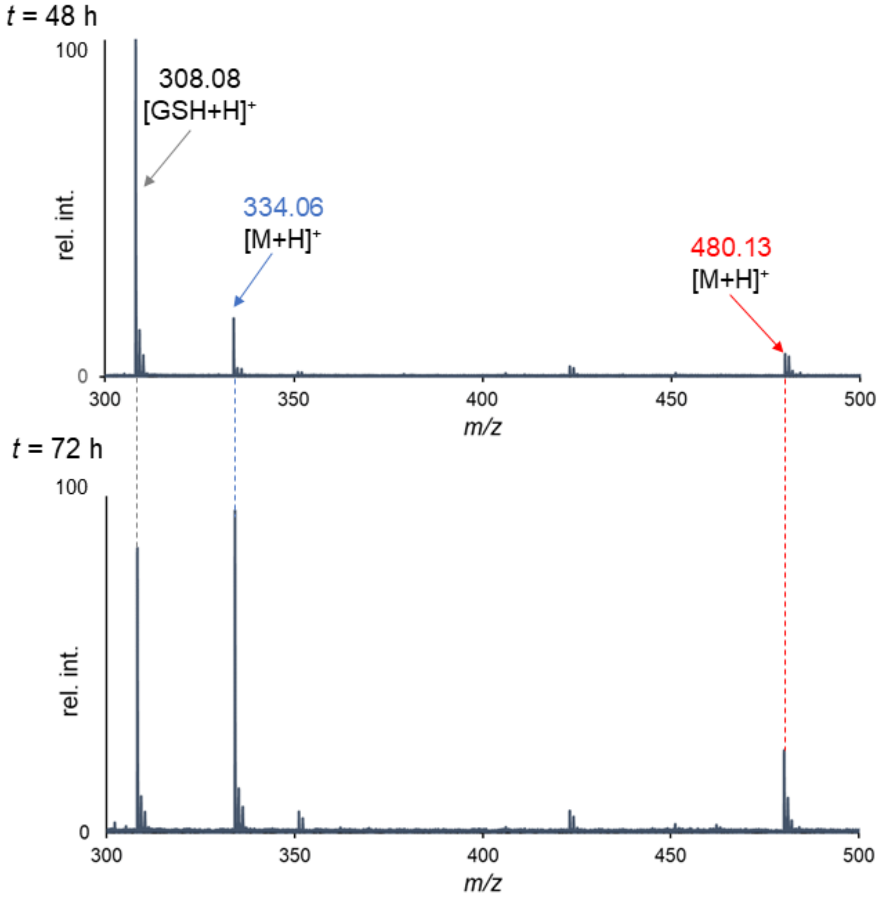

Figure S1. ESI-MS analysis of the reaction between 2-cyanopyrydine **1b** and glutathione.

The reaction between 2-cyanopyridine 1c and glutathione

Following the representative procedure described above, the progress of the reaction between 2-cyanopyridine **1c** and glutathione was monitored by ESI-MS analysis at 24 h and 72 h.

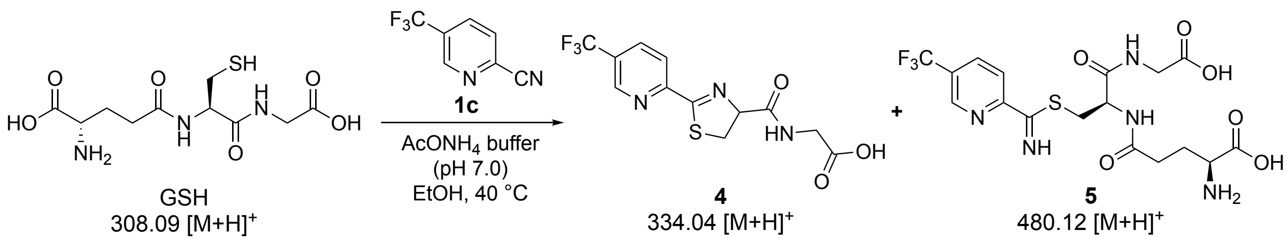

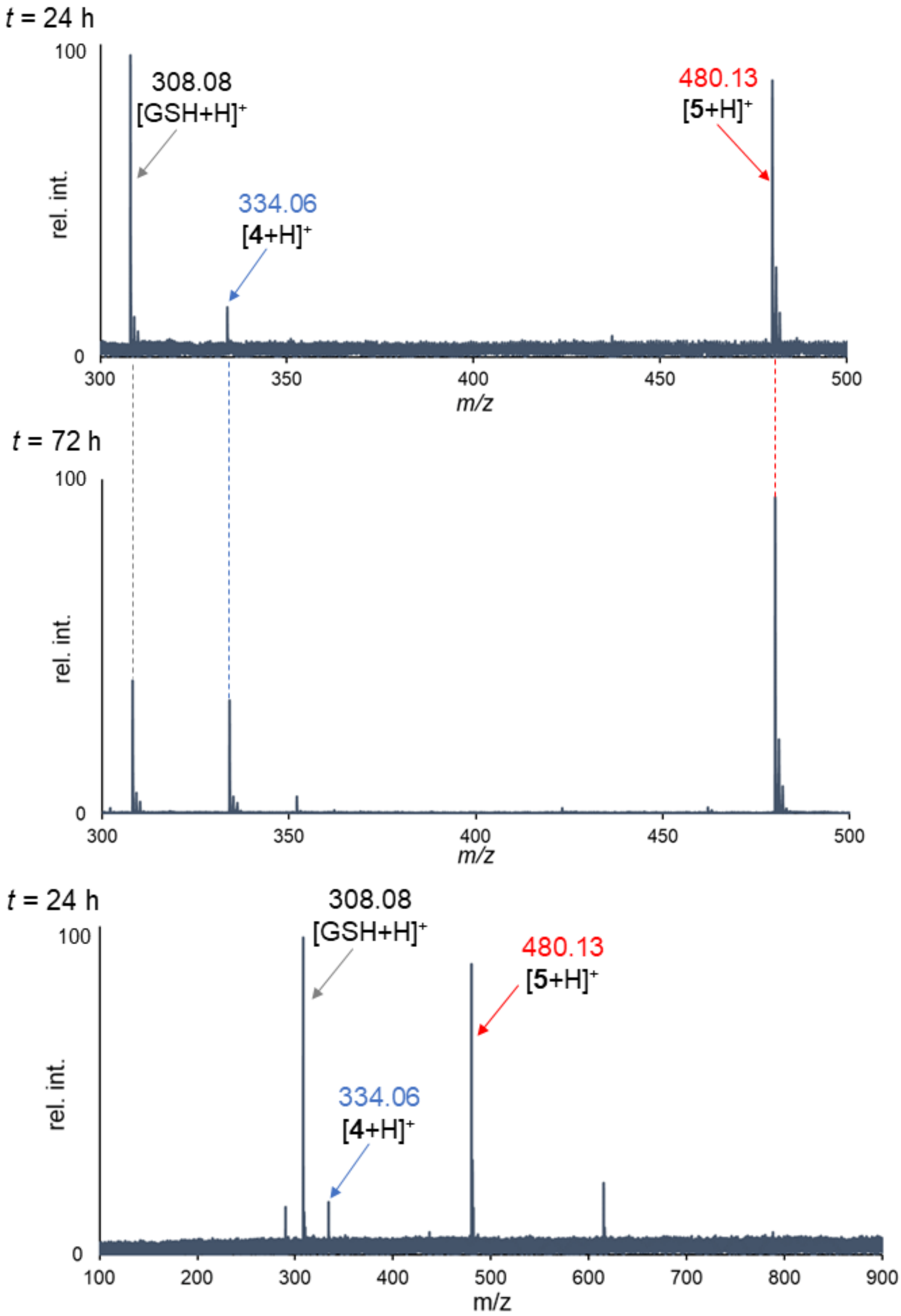

Figure S2. ESI-MS analysis of the reaction between 2-cyanopyrydine **1c** and glutathione.

The reaction between 2-cyanopyridine 1d and glutathione

Following the representative procedure described above, the progress of the reaction between 2-cyanopyridine **1d** and glutathione was monitored by ESI-MS analysis at 24 h and 96 h.

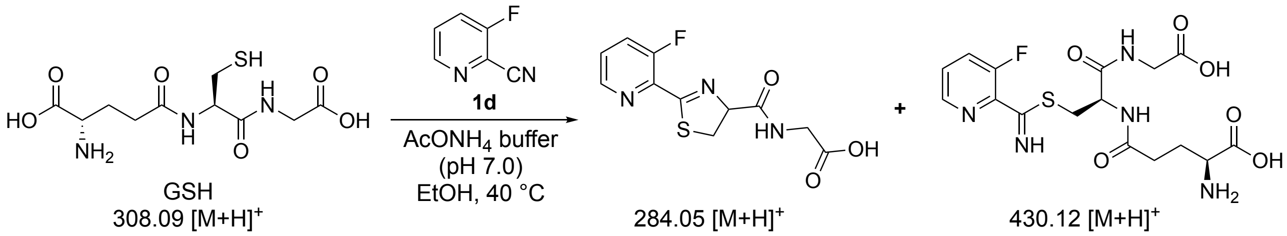

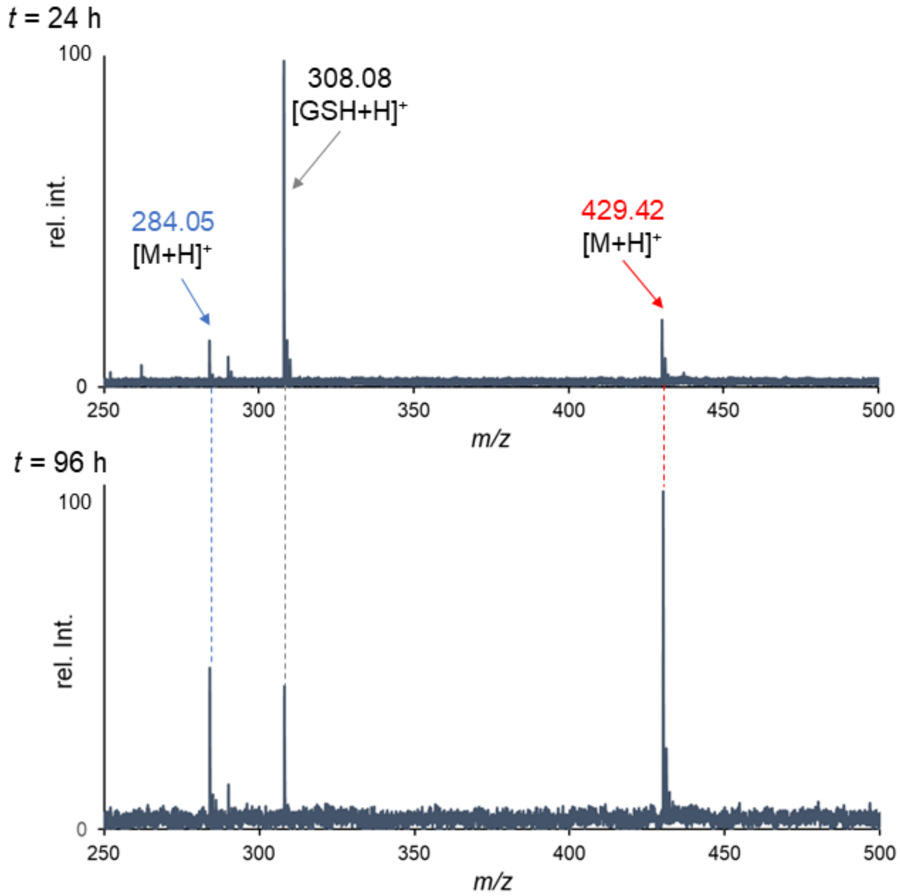

Figure S3. ESI-MS analysis of the reaction between **1d** and glutathione.

The reaction between 2-cyanopyridine 1e and glutathione

Following the representative procedure described above, the progress of the reaction between 2-cyanopyridine **1e** and glutathione was monitored by ESI-MS analysis at 48 h and 72 h.

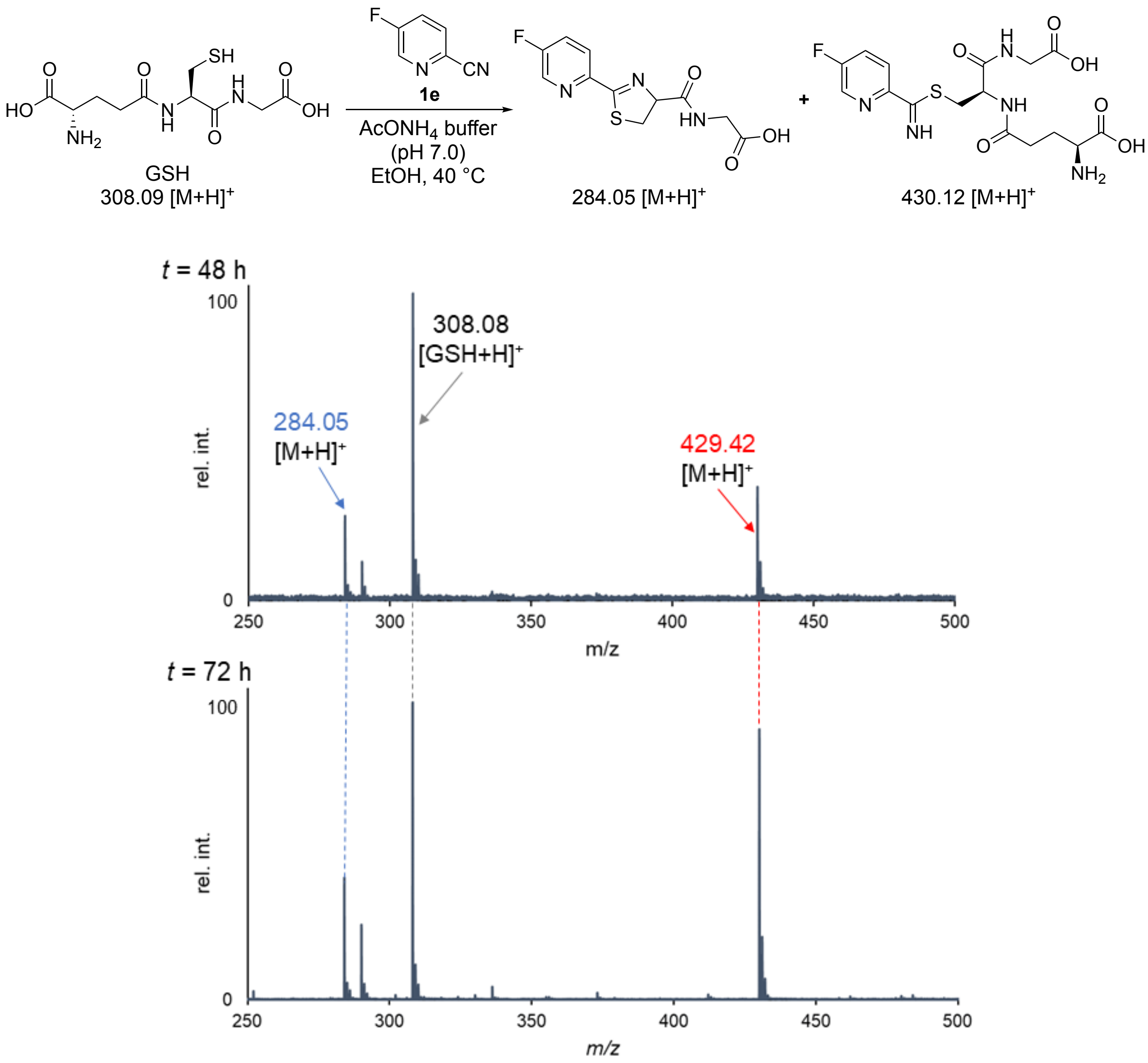

Figure S4. ESI-MS analysis of the reaction between 2-cyanopyridine **1e** and glutathione.

N-Terminal cysteine-bioconjugation of bioactive peptides

Bioactive peptides such as oxytocin (**6**), vasopressin (**9**), and lypressin (**12**) were purchased from commercial sources and used without further purification.

Analytical HPLC

Analytical RP-HPLC was carried out with a COSMOSIL 5C$_{18}$-AR-II packed column (4.6 × 250 mm). Bioconjugation efficiency was evaluated by analytical RP-HPLC, and all RP-HPLC was performed with a linear gradient of 10–40% acetonitrile and H_2O containing 0.1% (v/v) TFA over 30 min with a flow rate of 1.0 mL min^{-1} at room temperature. The eluting products were detected by UV at 220 nm and the mass spectra were acquired by ESI-MS in positive detection mode.

General procedure for the reaction between bioactive peptides and 2-cyanopyridine 1d

To a solution of peptide (1 mg, 1 µmol, 4.3 mM) in 0.5 M TCEP (pH 7.0) aqueous solution (62 µL, 134 mM) was added a solution of **1d** (1 mg, 8.2 µmol, 35 mM) in THF (10 µL) and water (160 µL). After stirring at 40 °C (silicone oil bath) for 1 h, 3–5 µL were withdrawn and dissolved in a 1:1 mixture of acetonitrile and H_2O containing 0.1% (v/v) TFA and analyzed by analytical RP- HPLC and ESI-MS. HPLC condition: 0.1% TFA (v/v) in water, 0.1% TFA (v/v) in acetonitrile, gradient 10-40% in 30 min, 1.0 mL min^{-1} flow rate, detected by UV at 220 nm.

The reaction between oxytocin (6) and 1d

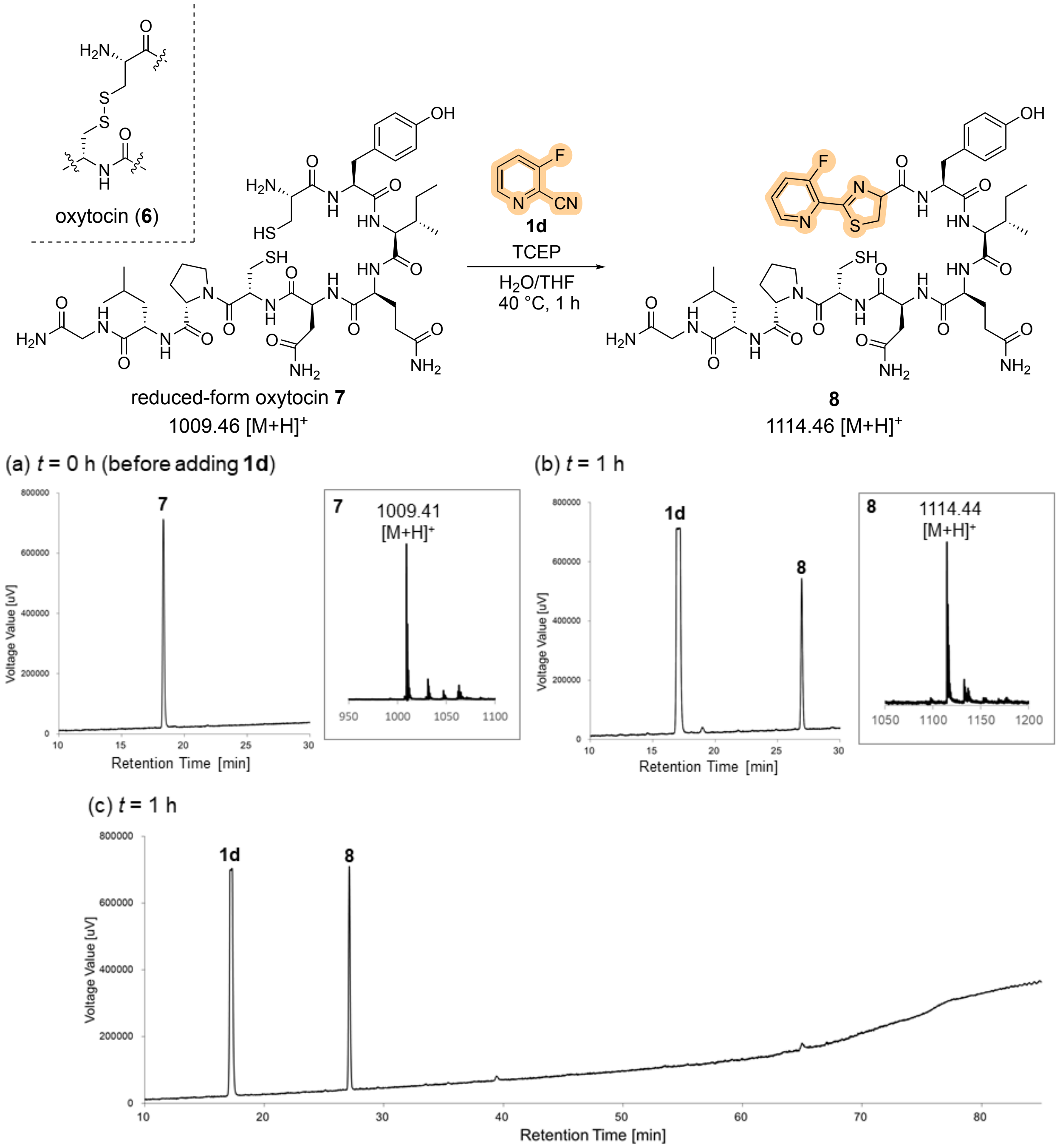

Figure S5. HPLC chart for the reaction of oxytocin (**6**) with 2-cyanopyridine **1d** at (a) $t = 0$ h (before adding **1d**) and (b) t = 1 h. The corresponding thiazoline product **8** was detected as a major HPLC peak; **8**, Retention time: 26.9 min; MS (ESI): m/z 1114.44 (calcd 1114.46 [M+H]$^+$). (c) HPLC chart

for a linear gradient of 10–95% acetonitrile and H_2O containing 0.1% (v/v) TFA over 85 min with a flow rate of 1.0 mL min^{-1}. The observed trace peaks were confirmed not to be derived from oxytocin.

The reaction between vasopressin (9) and 1d

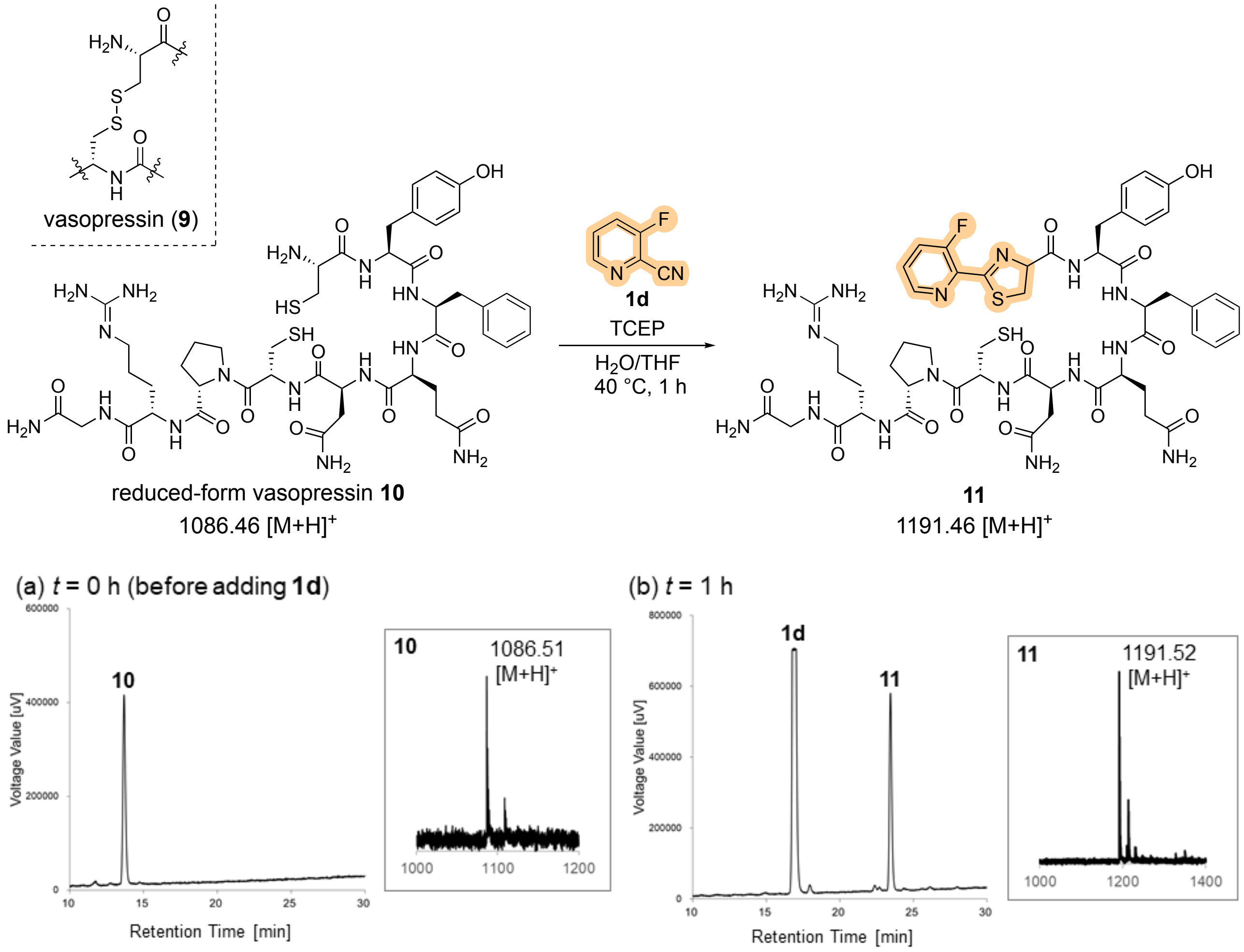

Figure S6. HPLC chart for the reaction of vasopressin (**9**) with 2-cyanopyridine **1d** at (a) t = 0 h (before adding **1d**) and (b) t = 1 h. The corresponding thiazoline product **11** was detected as a major HPLC peak; **11**, Retention time: 13.7 min; MS (ESI): *m/z* 1191.52 (calcd 1191.46 [M+H]$^+$)

The reaction between lypressin (12, lysine vasopressin) and 1d

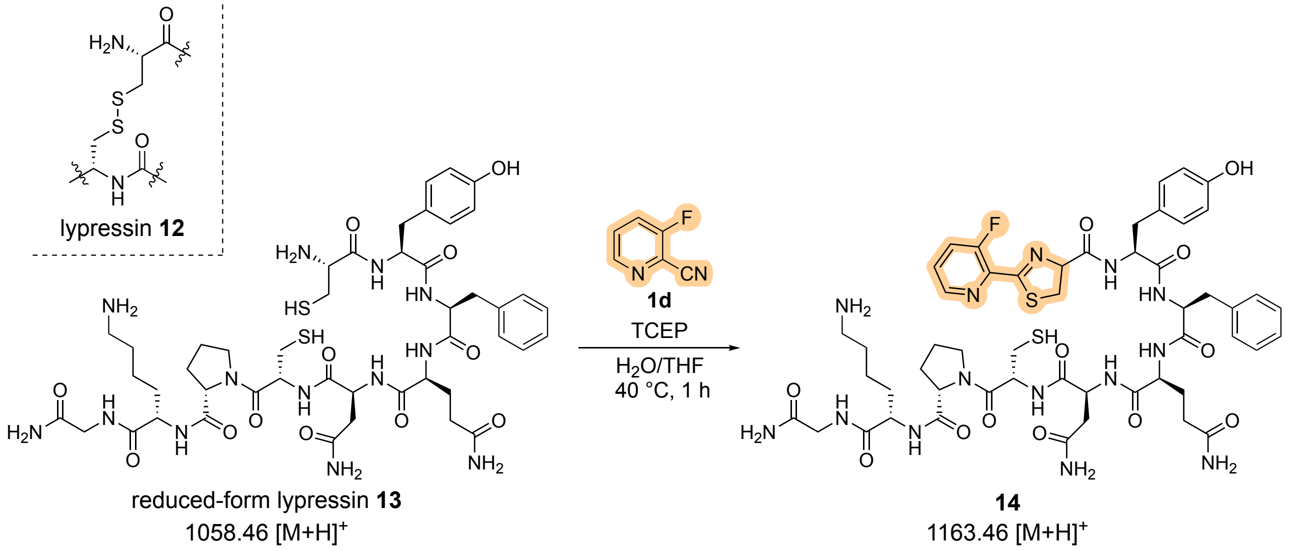

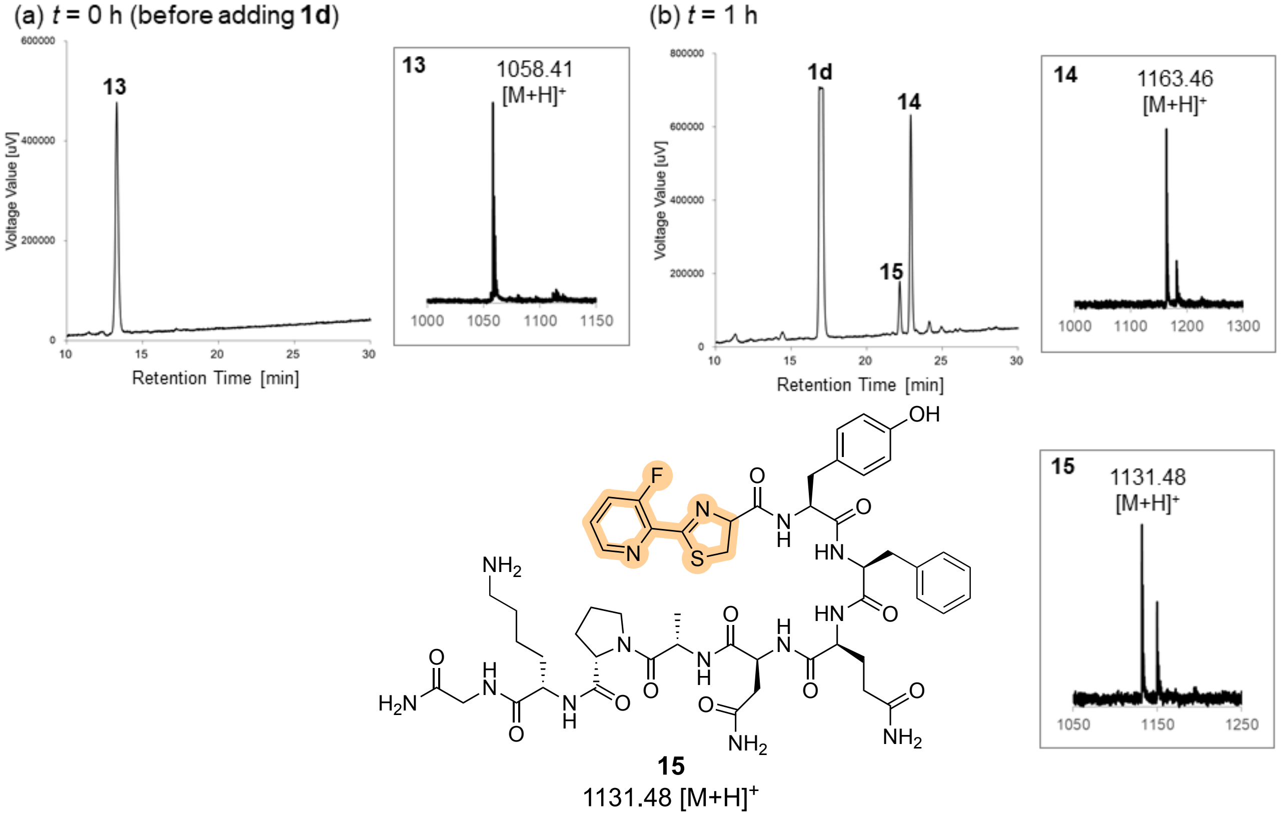

Figure S7. HPLC chart for the reaction of lypressin (**12**) with 2-cyanopyridine **1d** at (a) *t* = 0 h (before adding **1d**) and (b) t = 1 h. The corresponding thiazoline product **14** was detected as a major HPLC peak; **14**, Retention time: 22.9 min; MS (ESI): *m/z* 1163.46 (1163.46 calcd $[M+H]^+$). The desulphurization product **15** was detected as a minor peak; **15**, Retention time: 22.1 min; MS (ESI): *m/z* 1131.48 (1131.48 calcd $[M+H]^+$).

In the case of lypressin, the desulphurization product **15** was detected. This result indicates that the thiazoline products from bioactive peptides could be potentially unstable under the reductive conditions and the main decomposition pathway may be the desulphurization reaction.

^{15}N-Labeling experiments

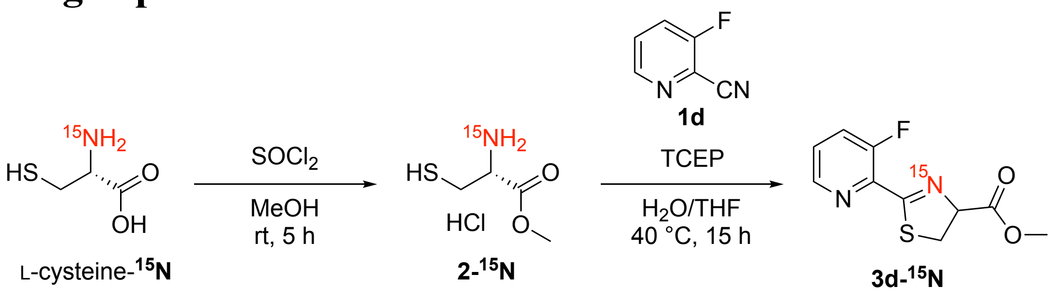

To a solution of L-cysteine-^{15}N (1.0 mg, 8.25 μmol) in dehydrated MeOH (66 μL) was added $SOCl_2$ (6 μL, 10 equiv.) at room temperature. After stirring at room temperature for 5 h, the reaction mixture was concentrated in *vacuo*. The resulting residue was dissolved in THF (16 μL) and 0.5 M TCEP (pH 7.0) aqueous solution (165 μL, 10 equiv.) and 3-fluoro-2-cyanopyridine **1d** (1.0 mg, 1.0 equiv.) was added. After stirring at 40 °C (silicone oil bath) for 15 h, the reaction was quenched with water. The aqueous layer was extracted with $CHCl_3$ and the combined organic layers were washed with brine, dried over $MgSO_4$, filtered and concentrated *in vacuo*. The crude product was purified by preparative RP-HPLC to afford the thiazoline product. Preparative RP-HPLC was performed with a Shim-pack PREP-ODS column (20 × 250 mm). HPLC condition: 0.1% TFA (v/v) in water, 0.1% TFA (v/v) in acetonitrile, 15% in 40 min, 10 mL min^{-1} flow rate, detected by UV at 254 nm. Product yield was not determined because the reaction scale was too small to allow accurate calculation. The entire obtained product was used for ^{15}N-NMR and HRMS experiments. ^{15}N-NMR and HRMS analysis confirmed that the thiazoline product **3d-^{15}N** contained ^{15}N-labeled nitrogen.

3d-^{15}N: ^{15}N NMR (30 MHz, $CDCl_3$) δ 307.8 (d, *J* = 14.3 Hz). HRMS (ESI) *m/z* calcd for $C_{10}H_7F^{15}N^{14}NO_2S+Na^+$: 264.0241 [M+Na]$^+$; found: 264.0237.

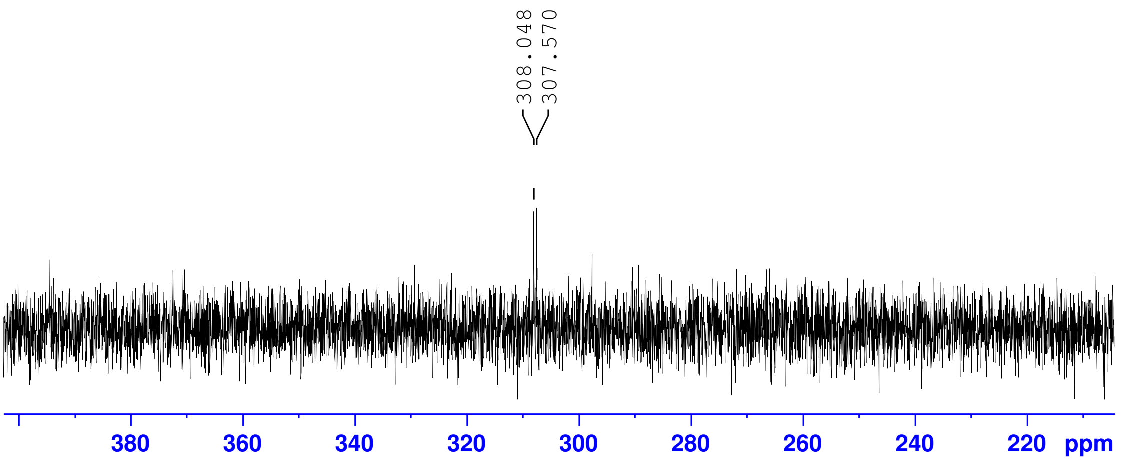

Figure S8. ^{15}N-NMR experiment of **3d-^{15}N** (30 MHz, $CDCl_3$).

Single-crystal X-ray diffraction analysis

The X-ray diffraction data of thiazoline product **3i** was collected by a Rigaku XtalLAB Synergy Custom (Custom-made machine). CCDC-2310511 for **3i** contain the supplementary crystallographic data for this paper. This data can be obtained free of charge from The Cambridge Crystallographic Data Centre via www.ccdc.cam.ac.uk/data_request/cif. and Fachinformationszentrum Karlsruhe Access Structures service.

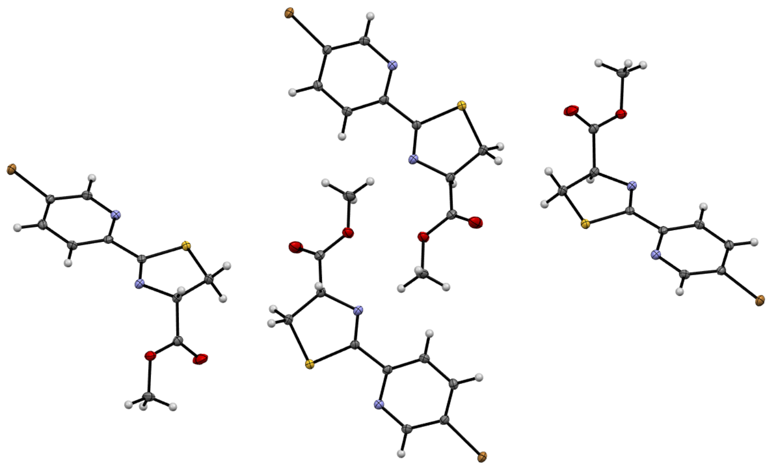

Figure S9. ORTEP view of thioether **3i**.

Crystal structure of compound 3i (CCDC 2310511)

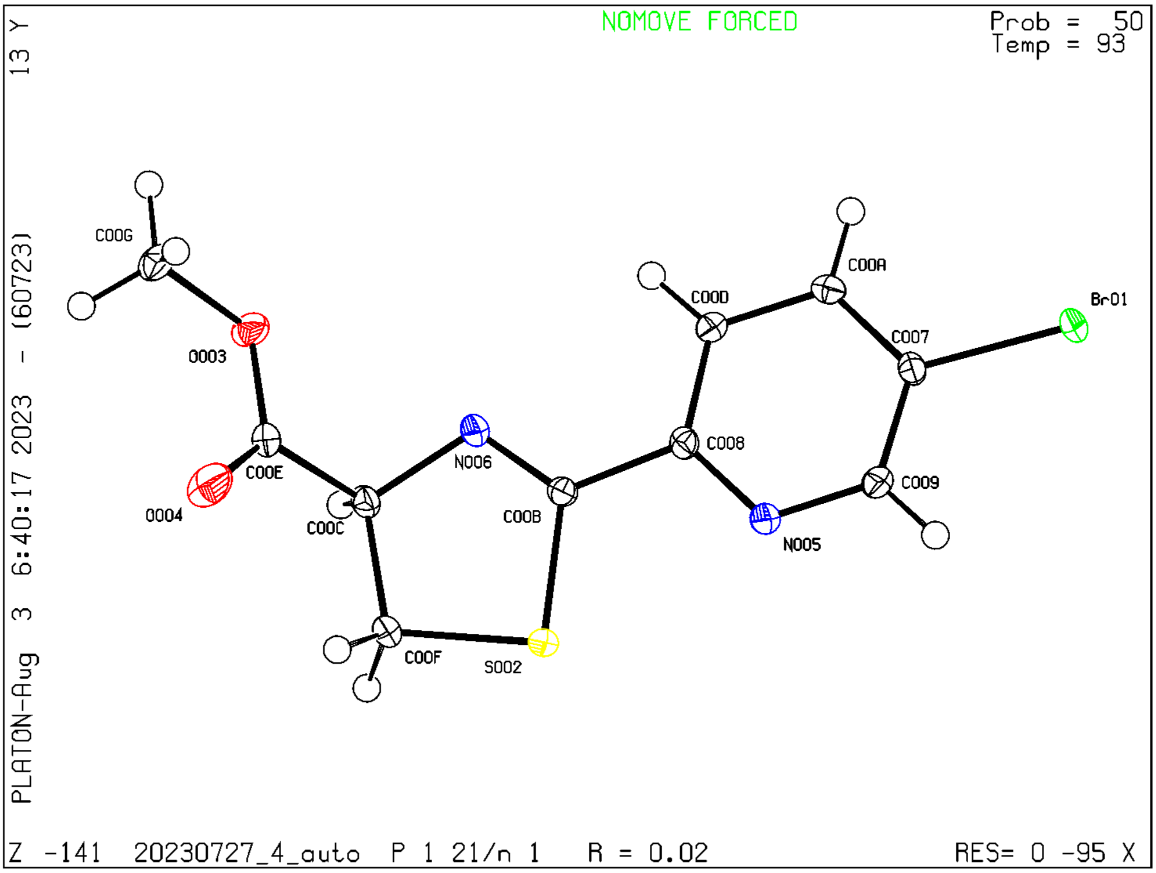

Thermal ellipsoid plot at the 50% probability level

Bond precision:	C-C = 0.0030 A		Wavelength=1.54184
Cell:	a=14.61601(13)	b=4.09391(4)	c=18.54407(16)
	alpha=90	beta=100.6459(8)	gamma=90
Temperature:	93 K		

	Calculated	Reported
Volume	1090.516(17)	1090.516(17)
Space group	P 21/n	P 1 21/n 1
Hall group	-P 2yn	-P 2yn
Moiety formula	C10 H9 Br N2 O2 S	C10 H9 Br N2 O2 S
Sum formula	C10 H9 Br N2 O2 S	C10 H9 Br N2 O2 S
Mr	301.15	301.16
Dx,g cm-3	1.834	1.834
Z	4	4
Mu (mm-1)	6.833	6.833
F000	600.0	600.0
F000’	599.94	
h,k,lmax	18,5,23	18,5,23
Nref	2309	2211
Tmin,Tmax	0.462,0.711	0.405,1.000
Tmin’	0.031	

Correction method= # Reported T Limits: Tmin=0.405 Tmax=1.000 AbsCorr = MULTI-SCAN

Data completeness= 0.958 Theta(max)= 77.250

R(reflections)= 0.0218(2205) wR2(reflections)= 0.0563(2211)

S = 1.132 Npar= 146

CD spectra of thiazoline products

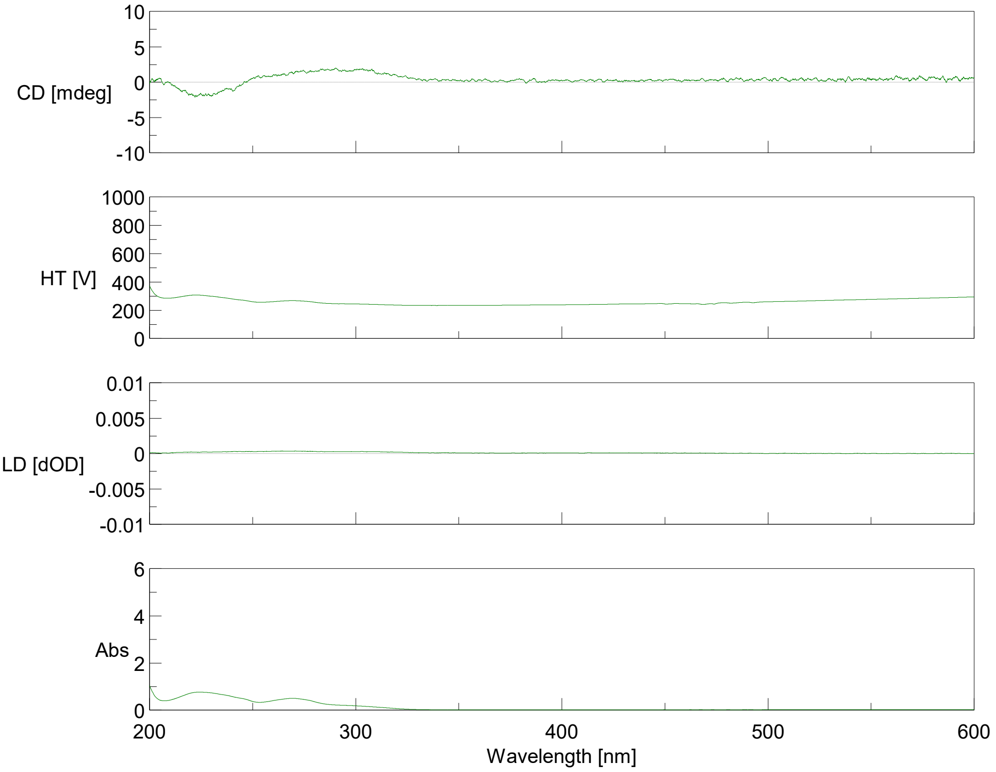

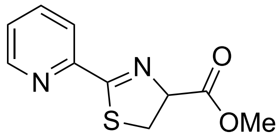

3a

Figure S10. CD spectra of thiazoline **3a** (621 μmol/L in MeOH).

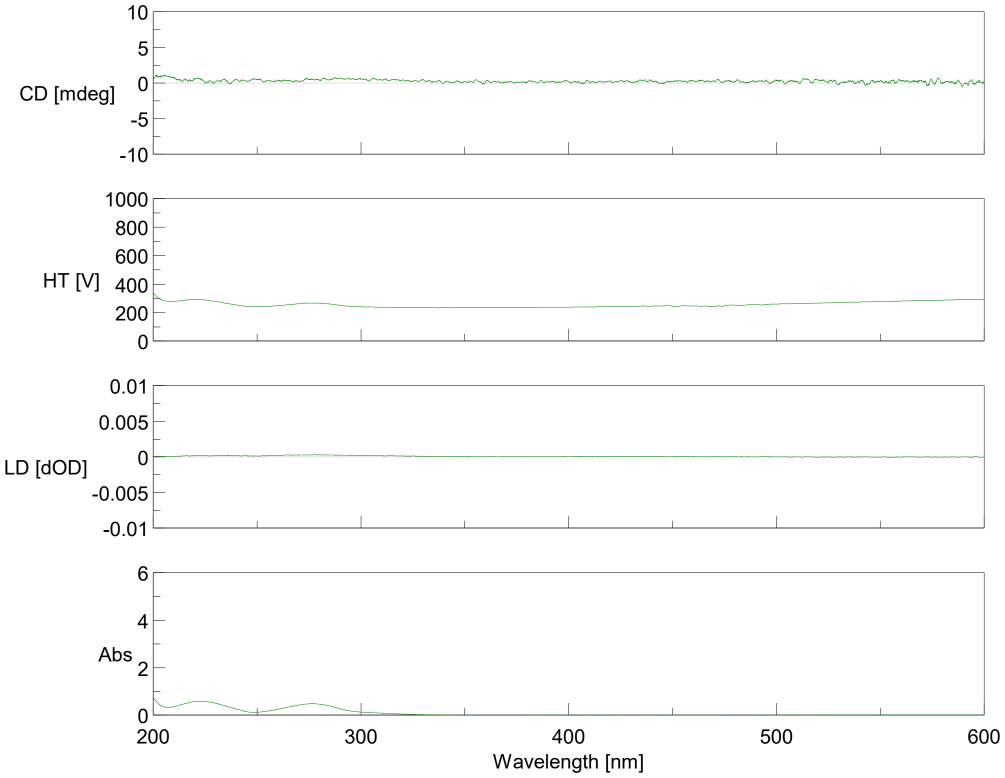

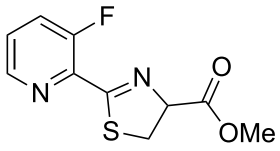

3d

Figure S11. CD spectra of thiazoline **3d** (570 μmol/L in MeOH).

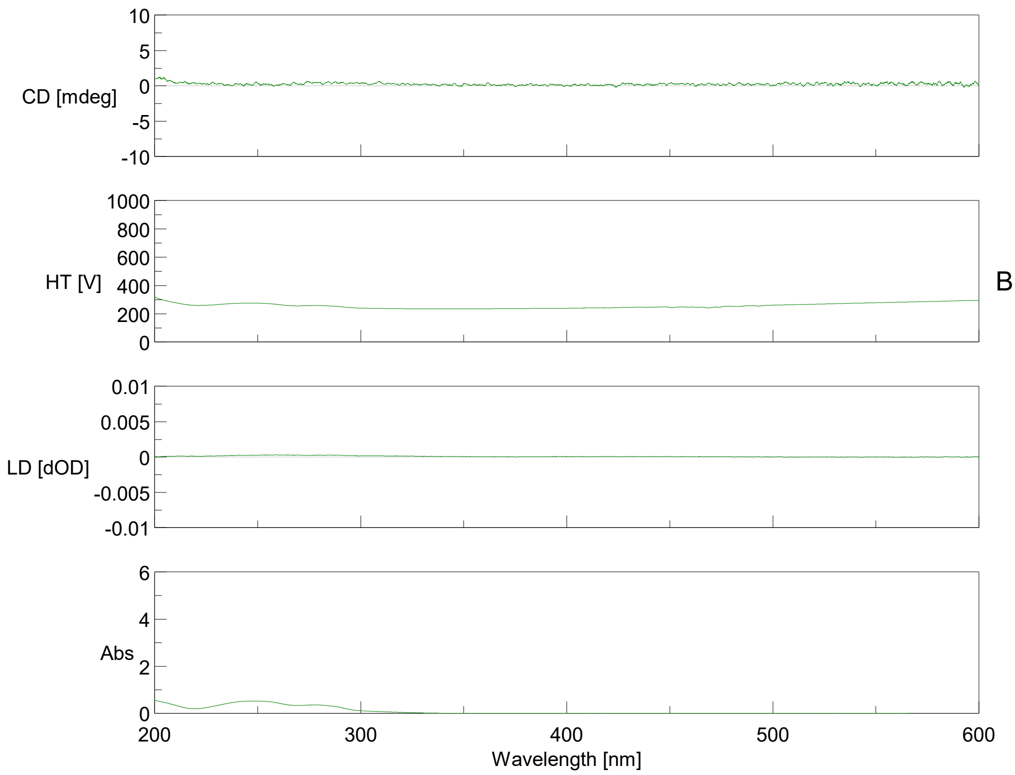

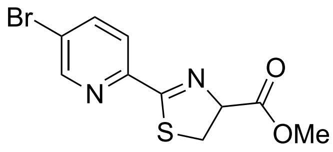

3i

Figure S12. CD spectra of thiazoline **3i** (352 μmol/L in MeOH).

No optical activity was detected in the CD spectrum of thiazolines **3a**, **3d**, and **3i**, indicating that the thiazoline products racemize very rapidly.

References

1) Gaillard, P.; Quattropani, A.; Pomel, V.; Rueckle, T.; Klicic, J.; Church, D. WO2007023186A1, 2007.

2) Cserép, G. B.; Demeter, O.; Bätzner, E.; Kállay, M.; Wagenknecht, H.-A.; Kele, P. *Synthesis* **2015**, *47*, 2738.

^{1}H-NMR of **1h** (300 MHz, $CDCl_3$)

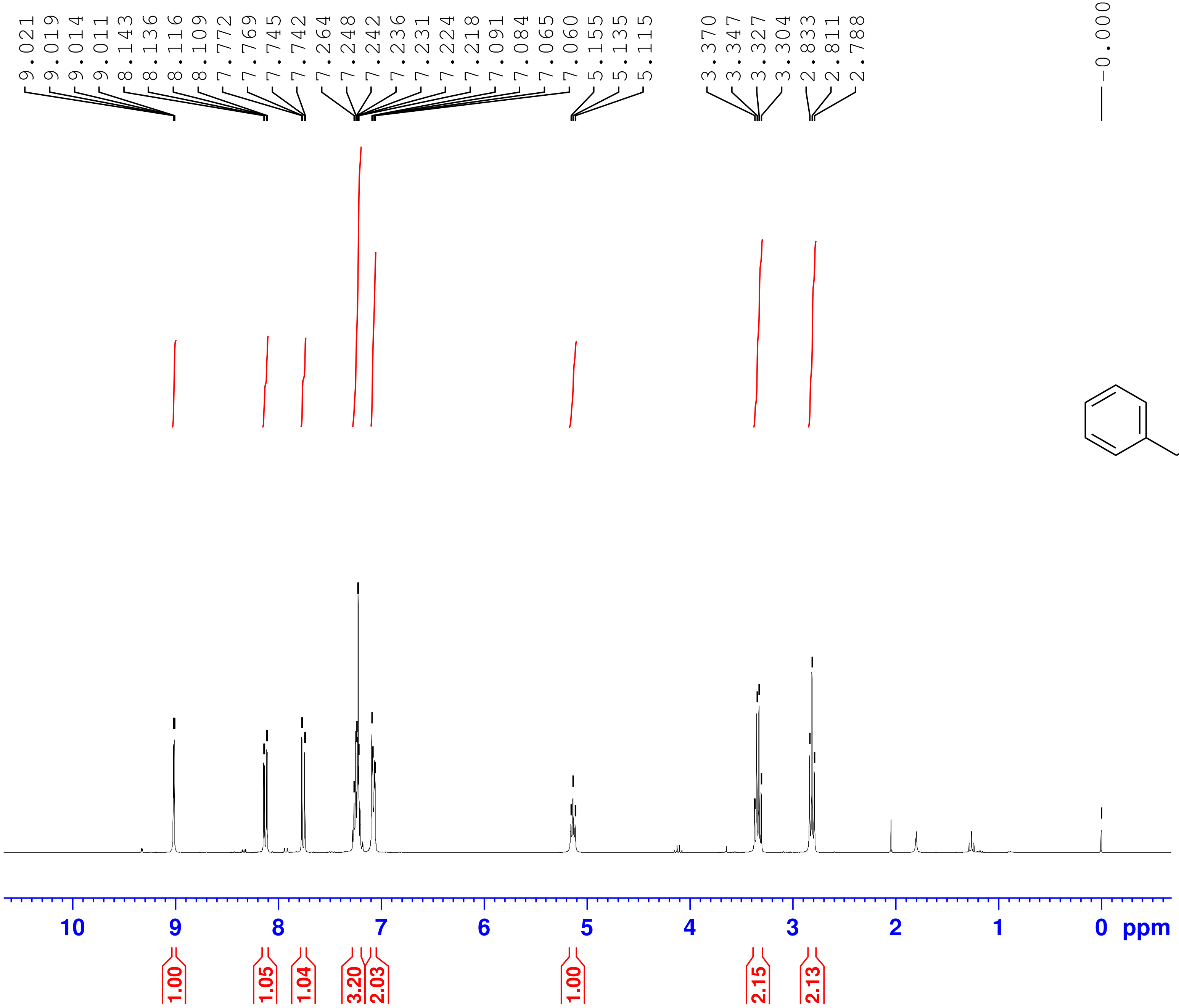

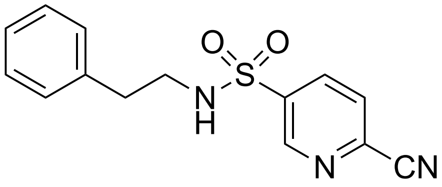

1h

^{13}C-NMR of **1h** (75 MHz, $CDCl_3$)

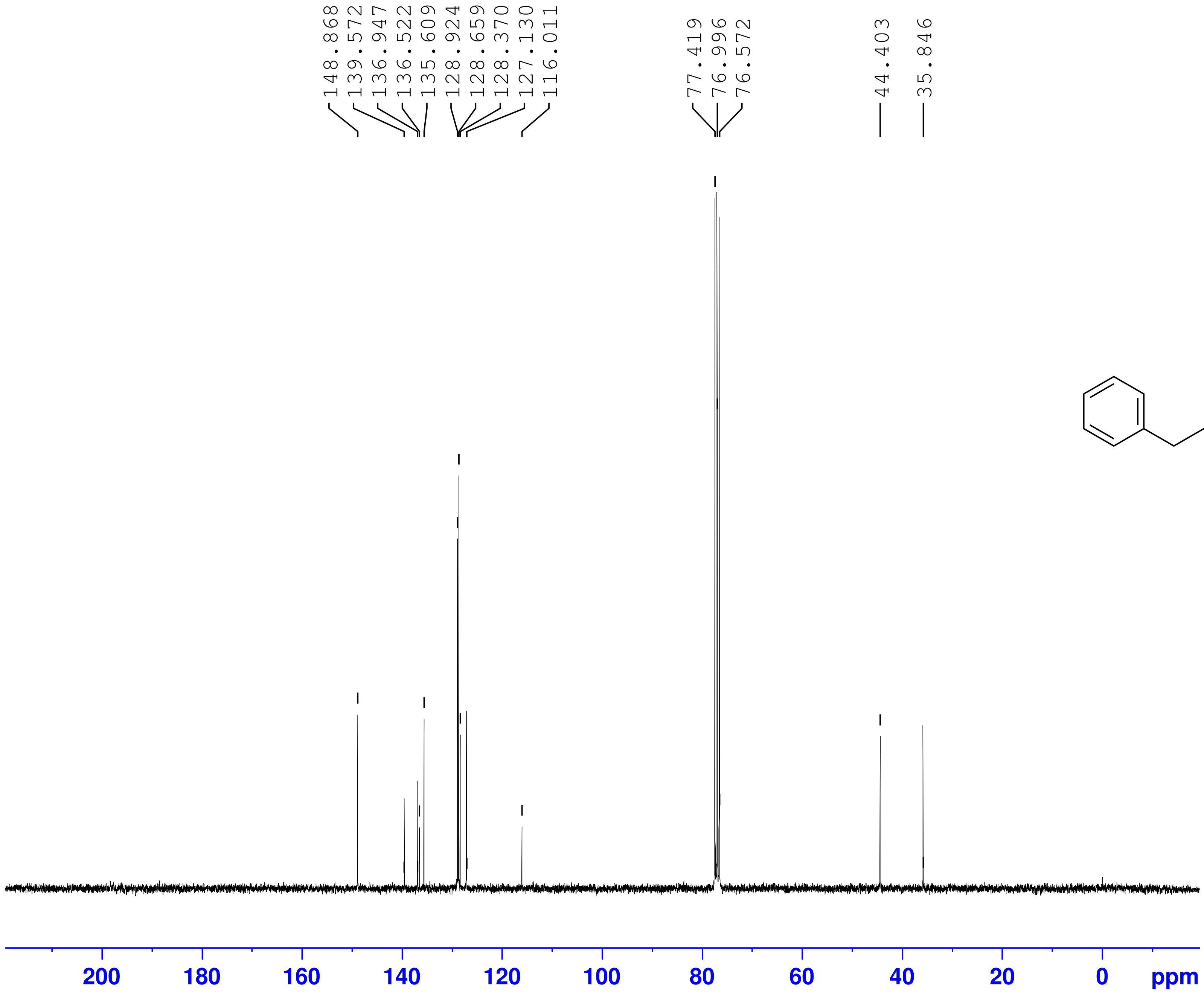

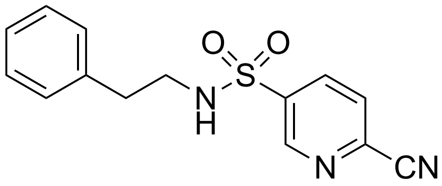

1h

^{1}H-NMR of **3a** (400 MHz, $CDCl_3$)

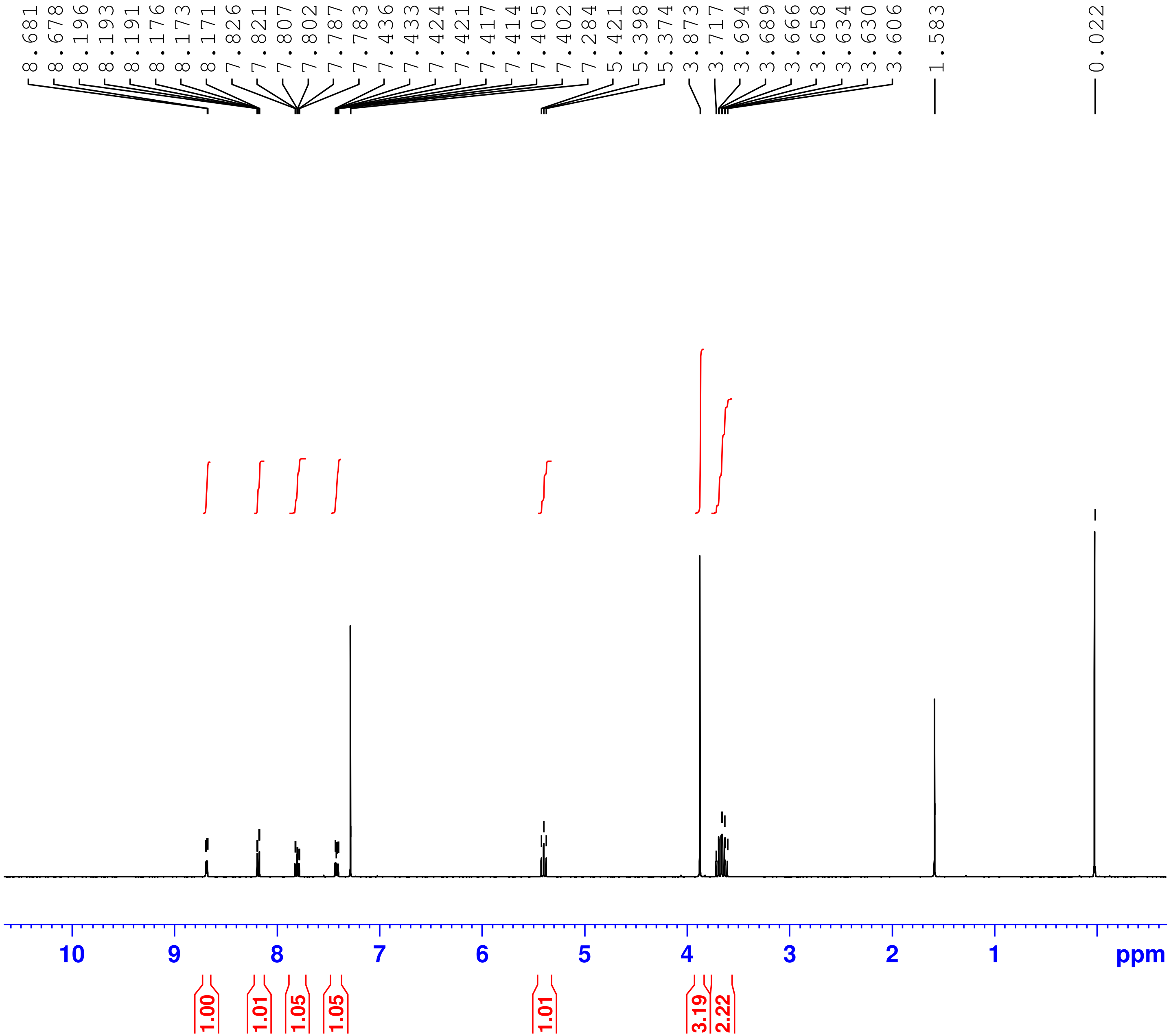

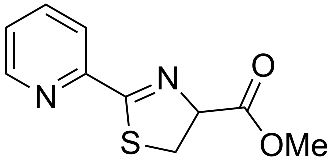

3a

^{13}C-NMR of **3a** (100 MHz, $CDCl_3$)

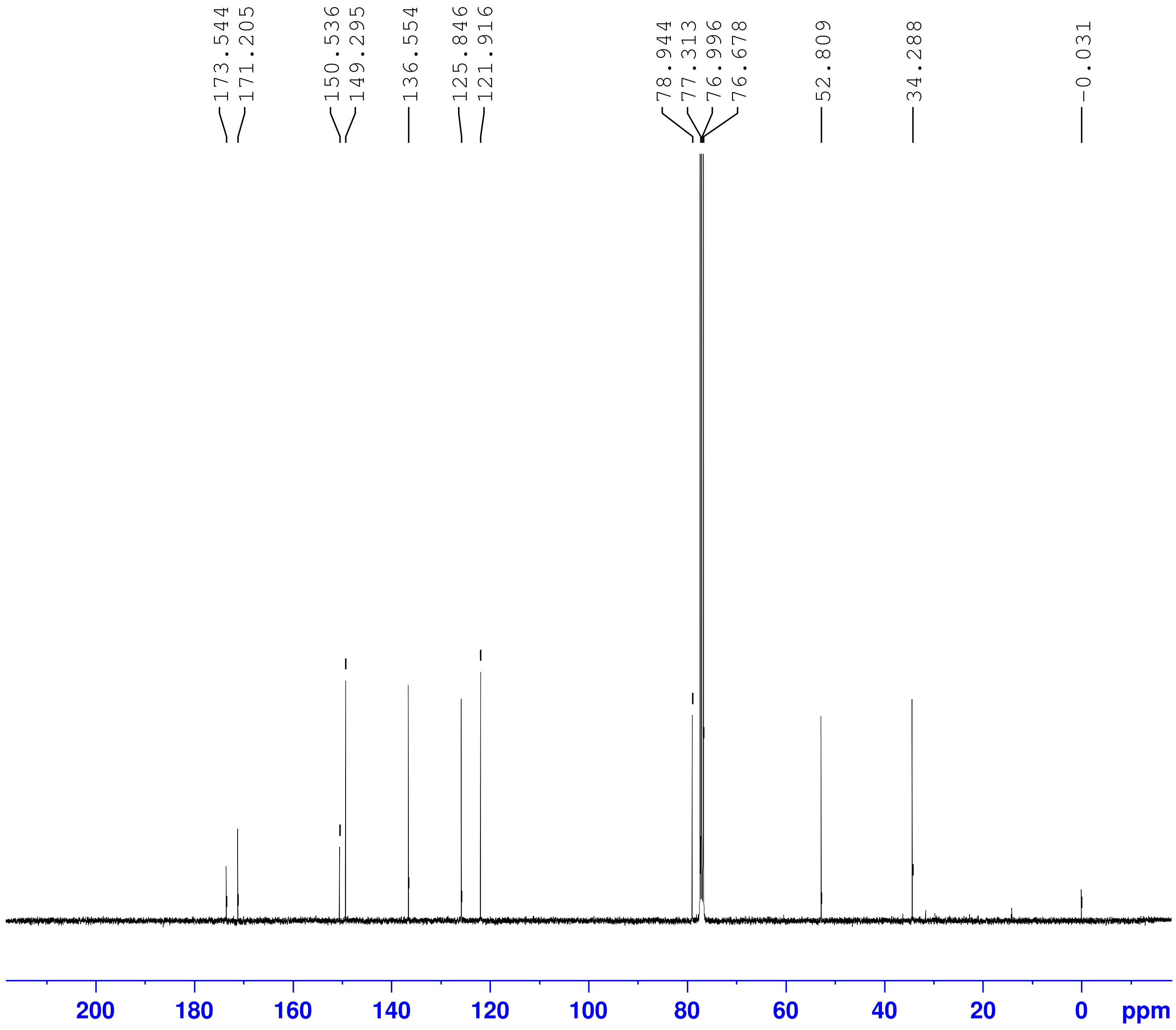

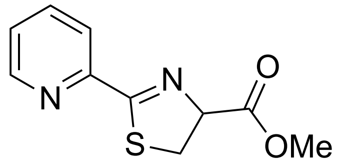

3a

^{1}H-NMR of **3b** (300 MHz, $CDCl_3$)

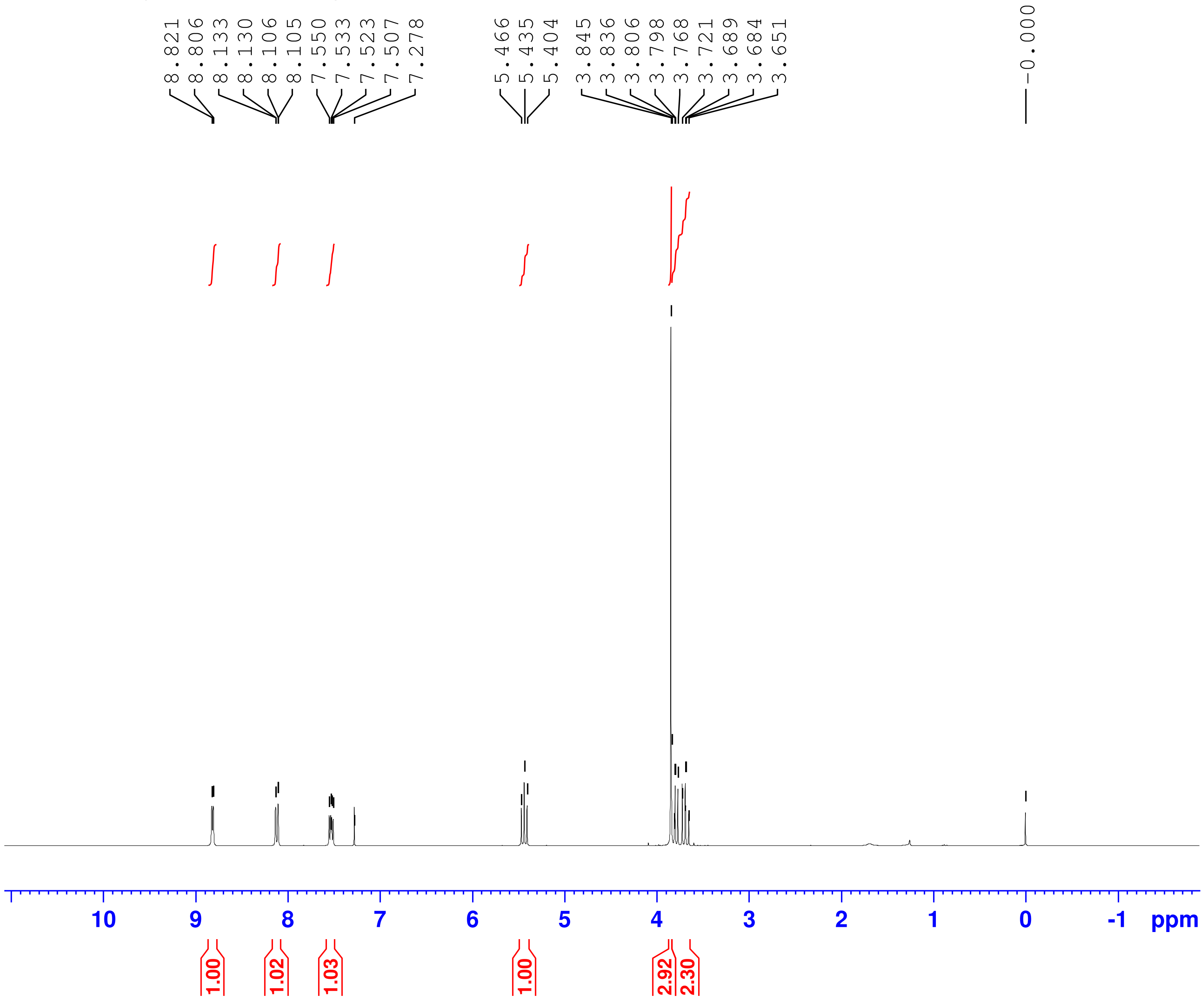

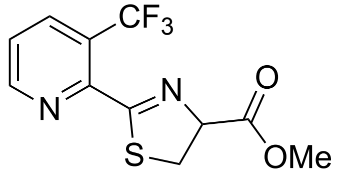

3b

^{13}C-NMR of **3b** (75 MHz, $CDCl_3$)

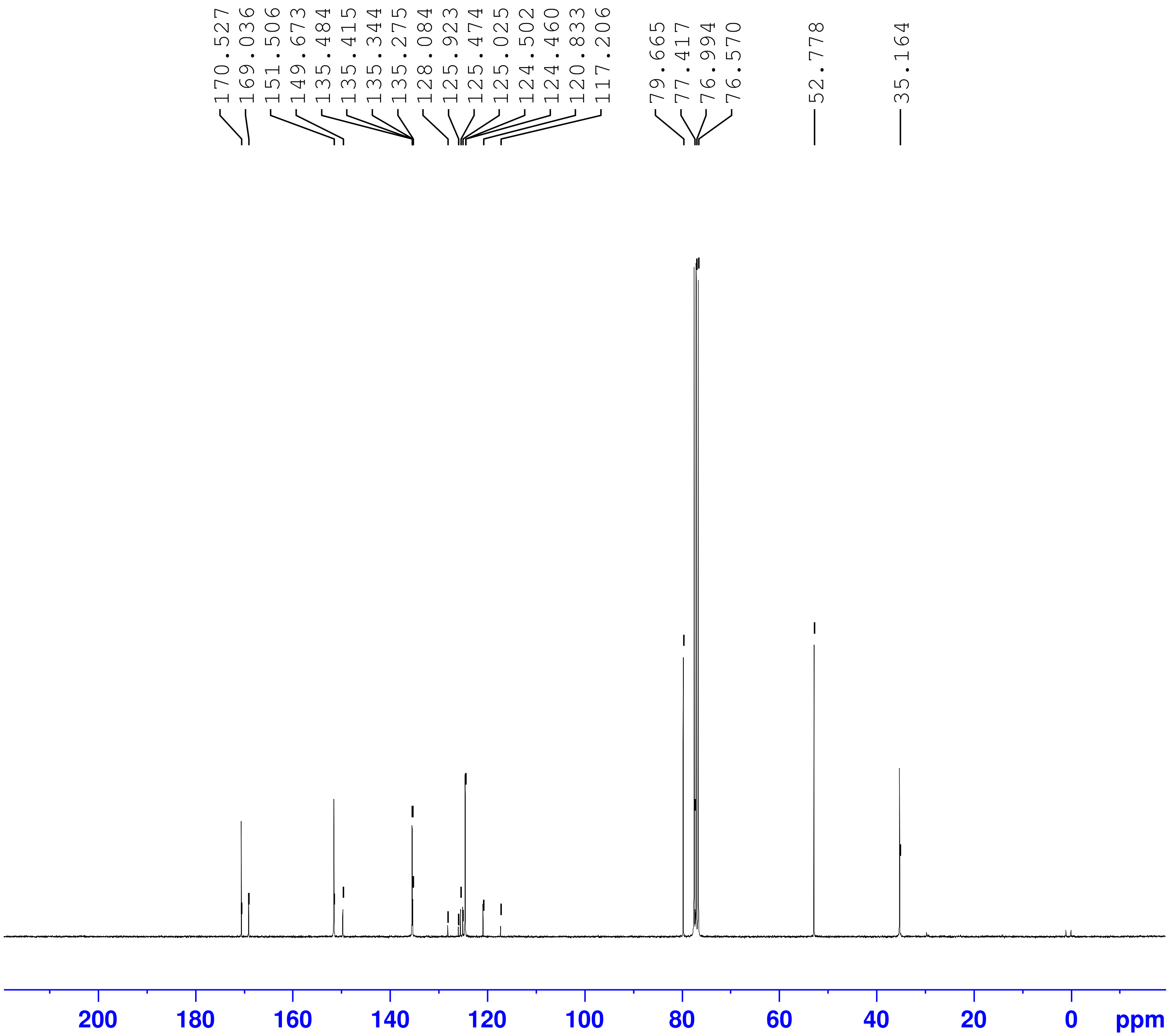

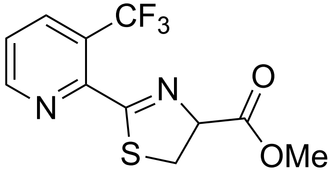

3b

^{1}H-NMR of **3c** (400 MHz, $CDCl_3$)

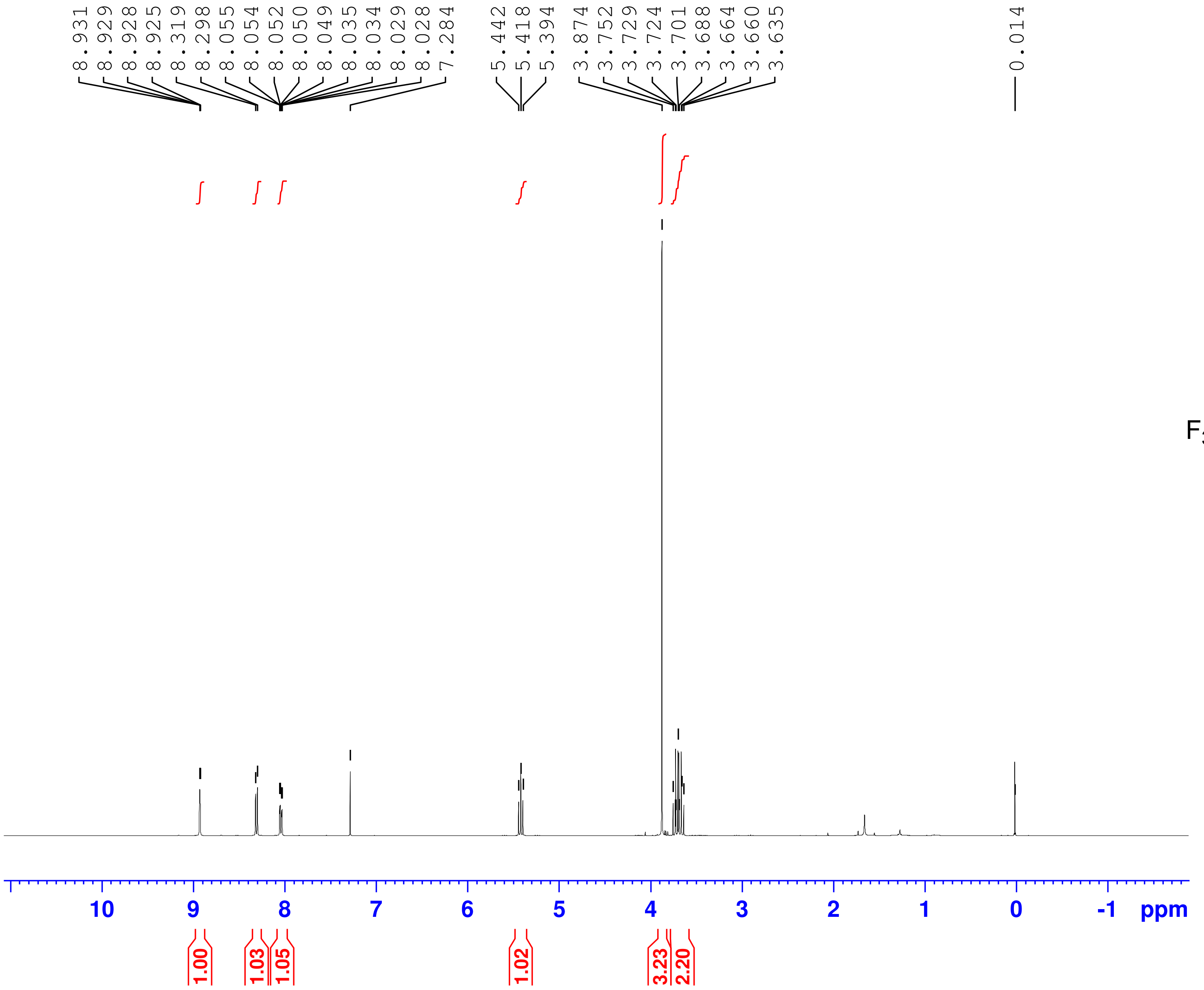

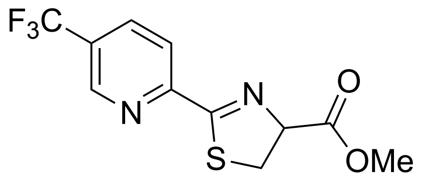

3c

^{13}C-NMR of **3c** (75 MHz, $CDCl_3$)

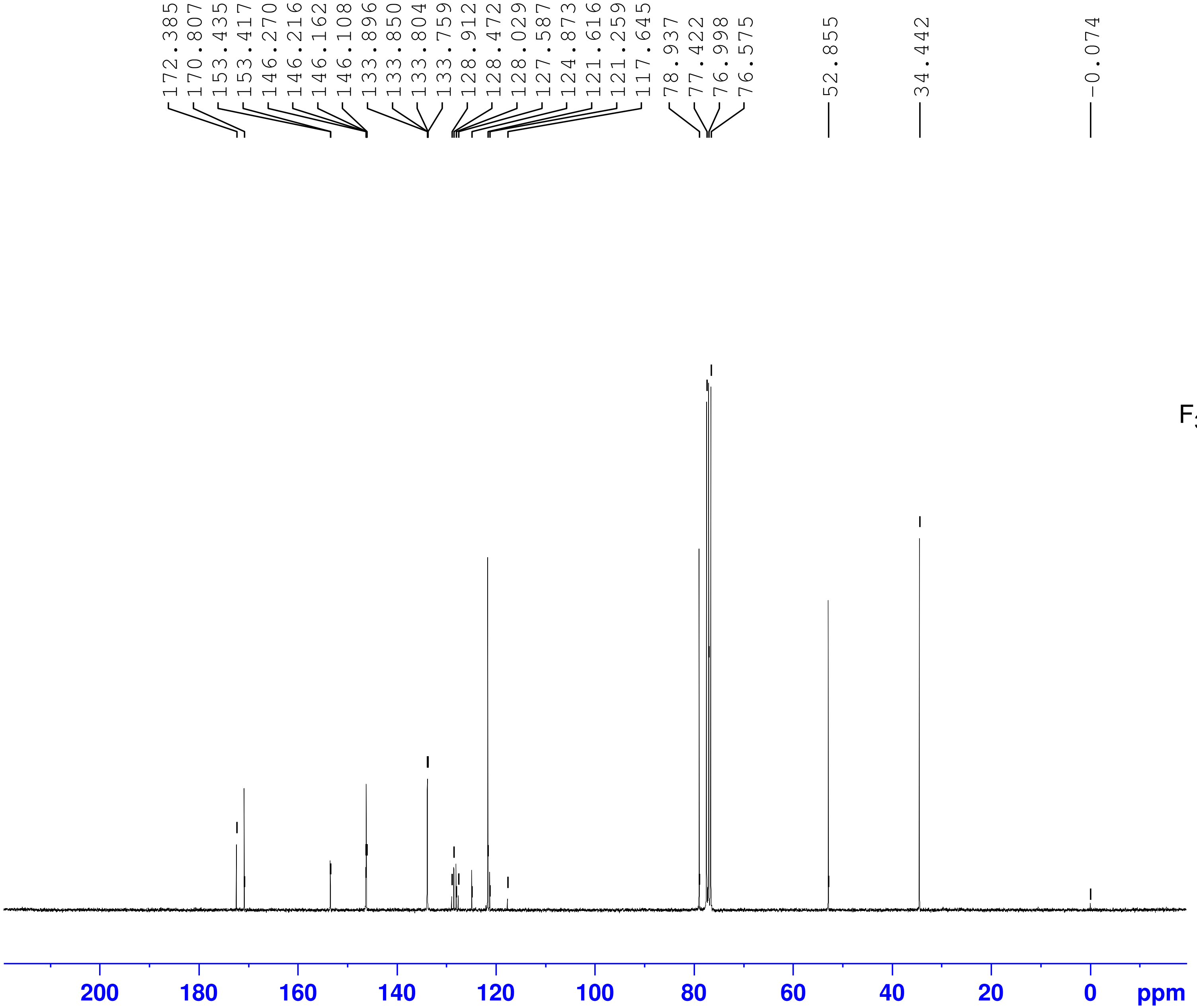

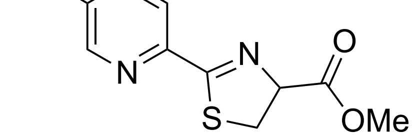

3c

^{1}H-NMR of **3d** (300 MHz, $CDCl_3$)

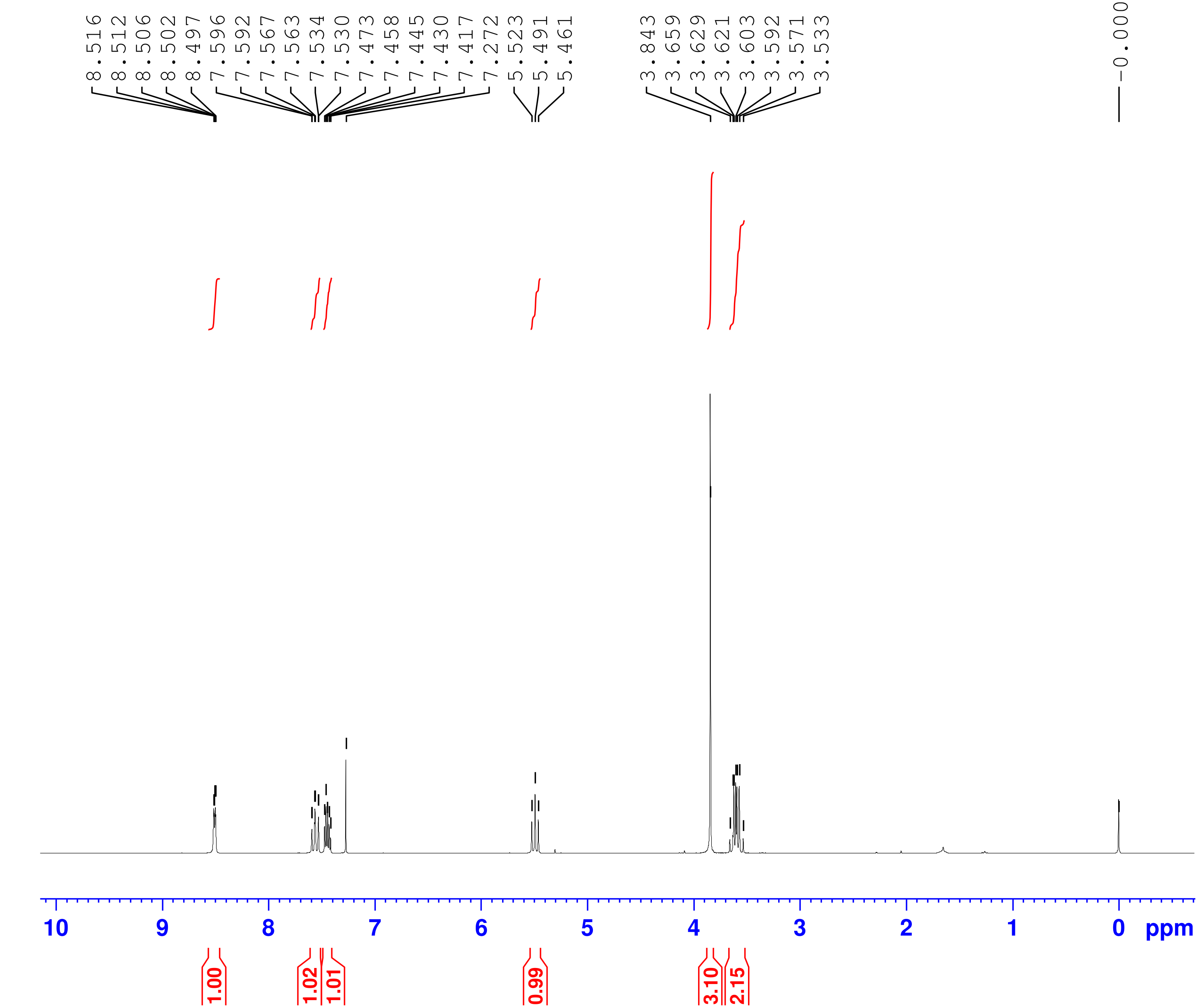

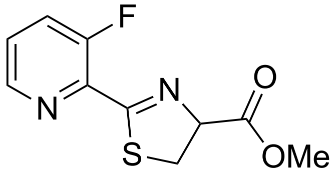

3d

^{13}C-NMR of **3d** (75 MHz, $CDCl_3$)

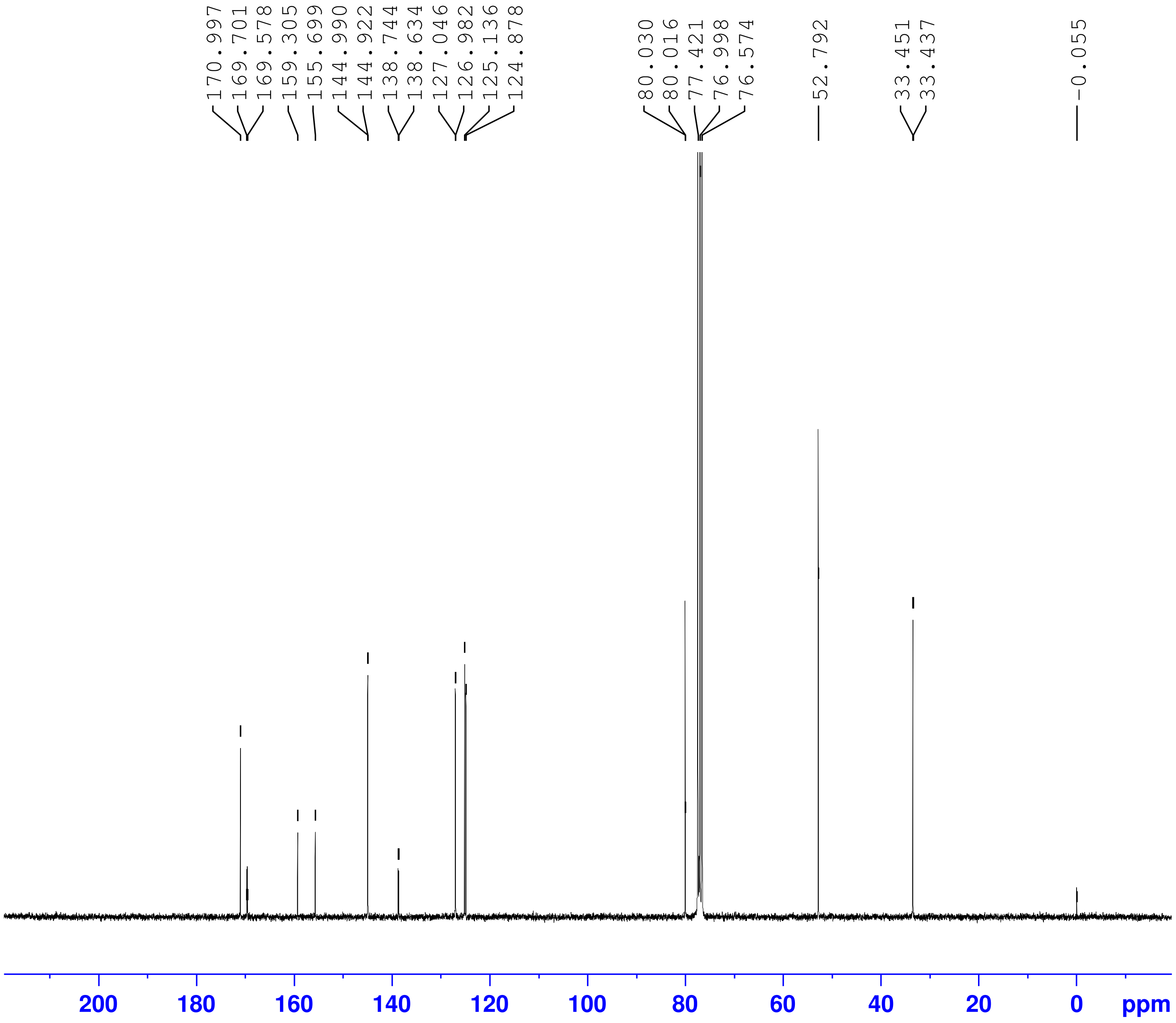

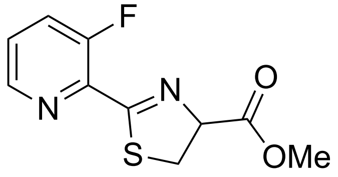

3d

^{1}H-NMR of **3e** (300 MHz, $CDCl_3$)

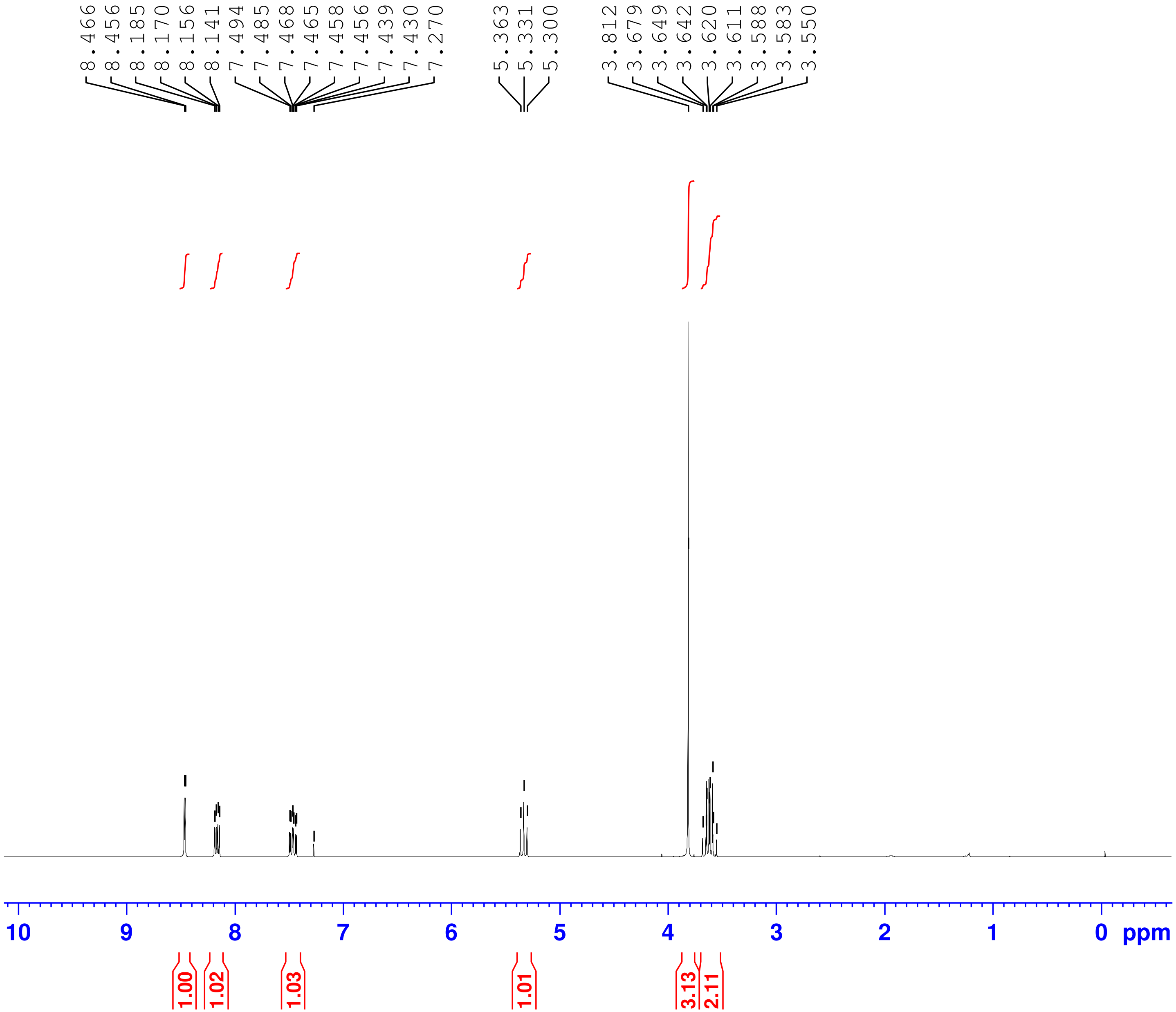

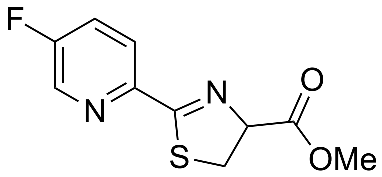

3e

^{13}C-NMR of **3e** (100 MHz, $CDCl_3$)

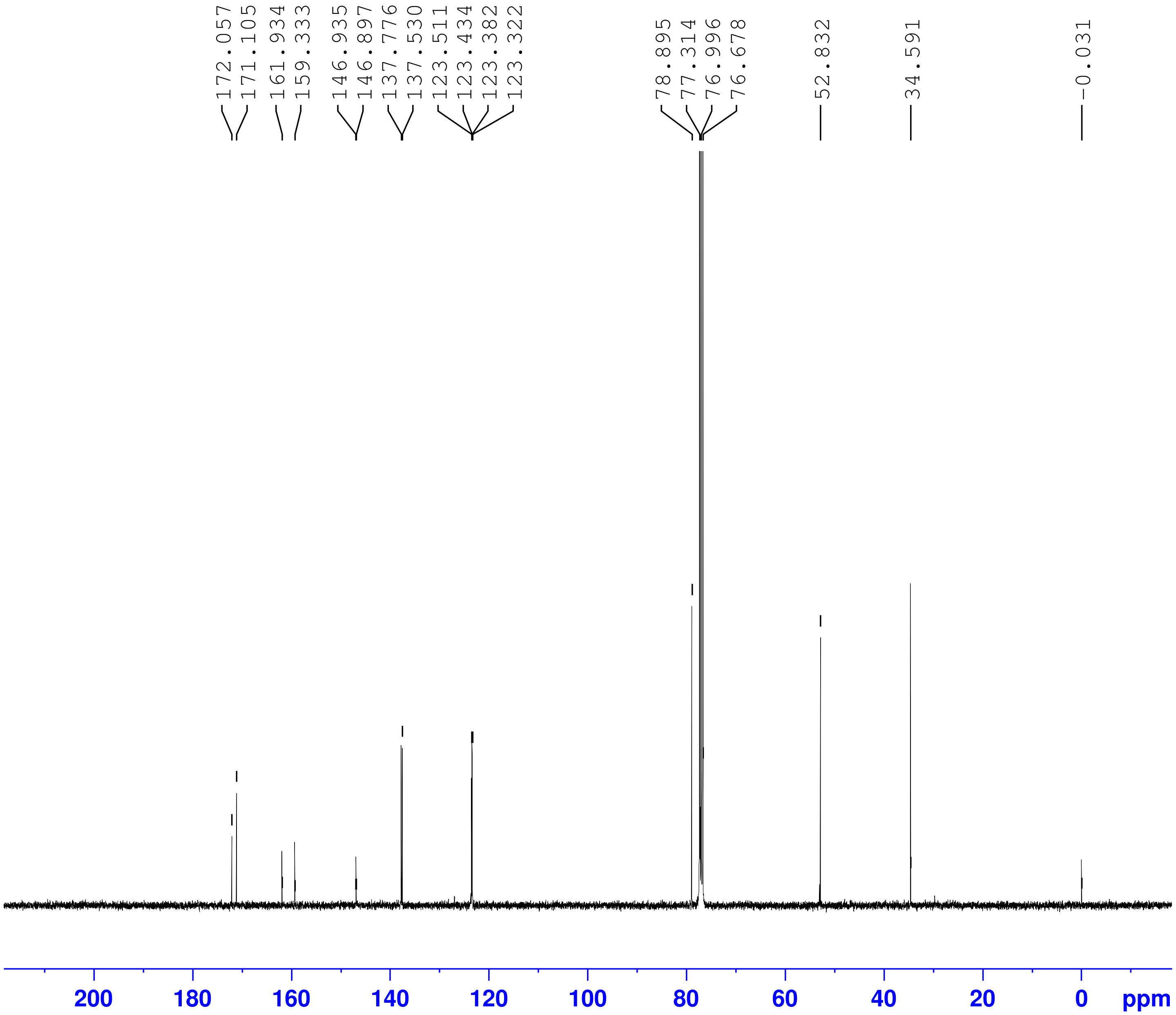

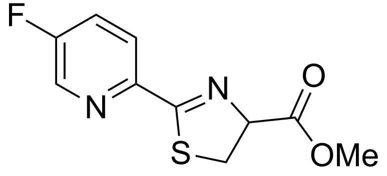

3e

^{1}H-NMR of **3g** (400 MHz, DMSO-d_6)

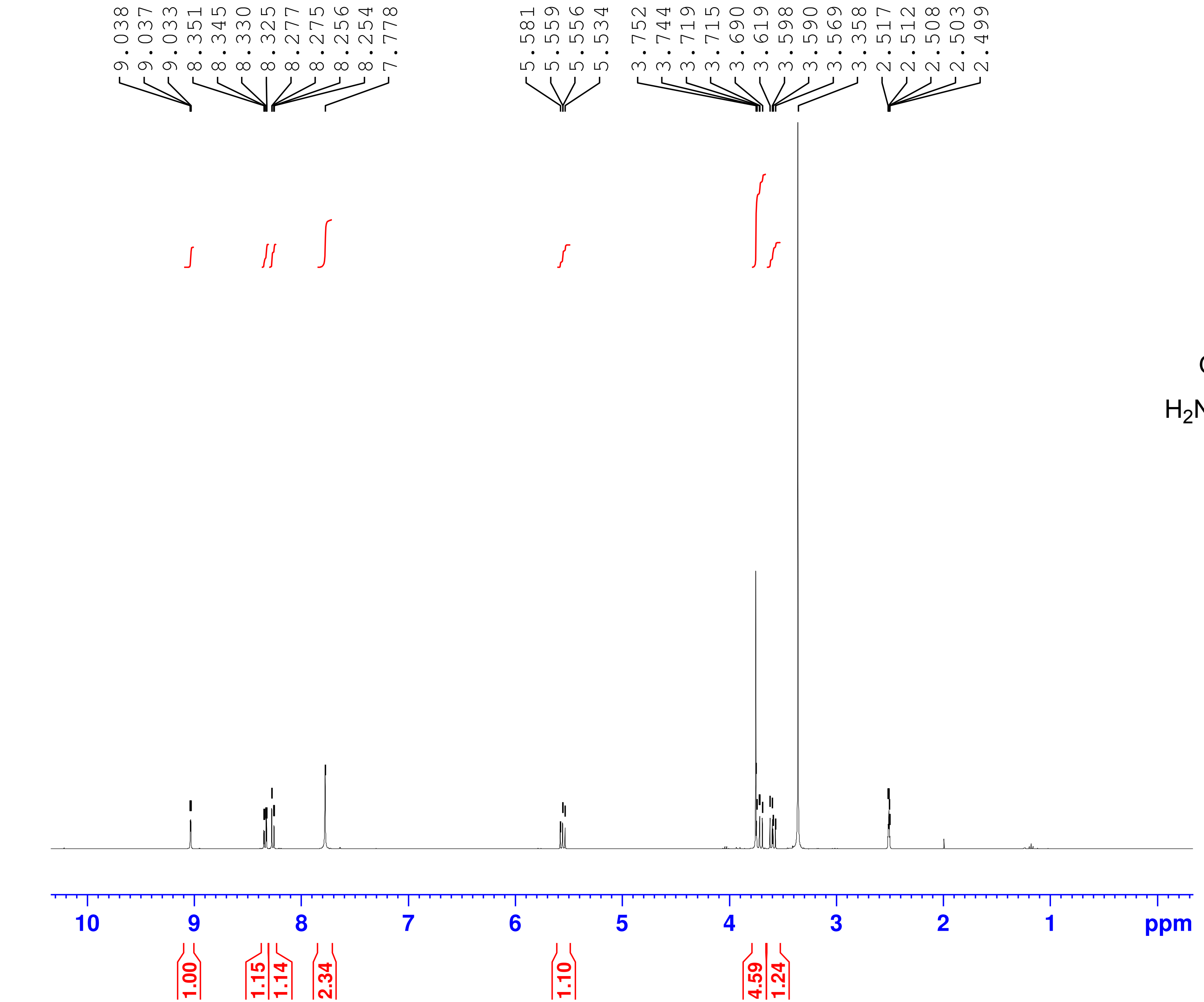

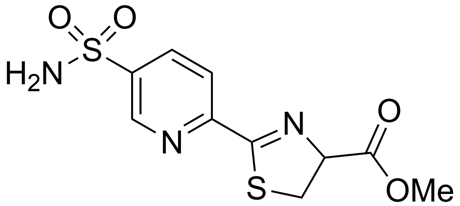

3g

^{13}C-NMR of **3g** (100 MHz, DMSO-d_6)

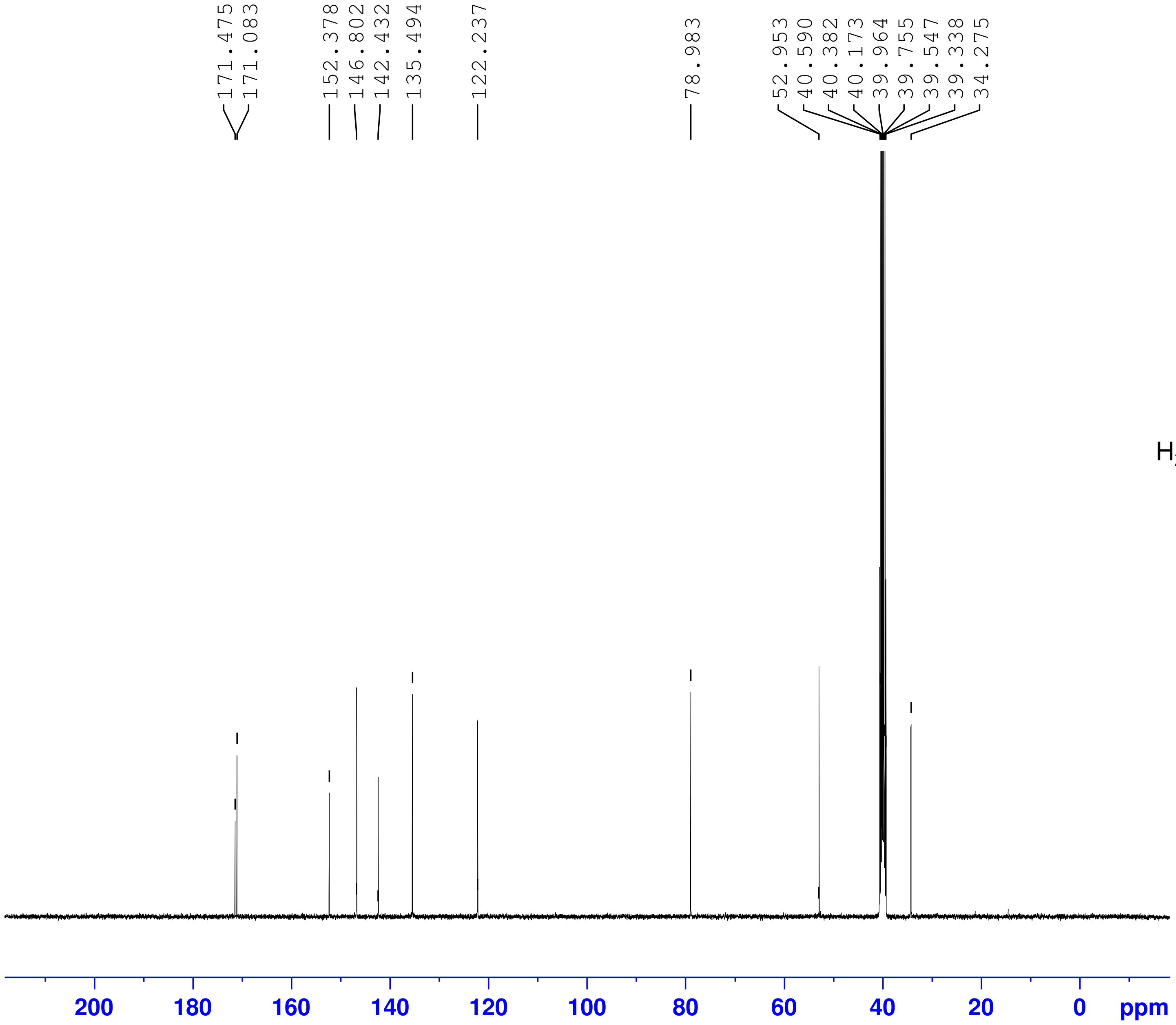

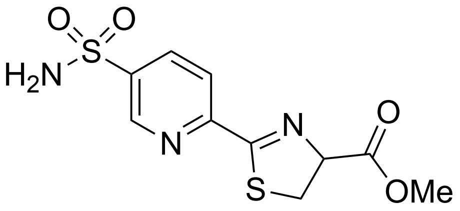

3g

^{1}H-NMR of **3h** (400 MHz, $CDCl_3$)

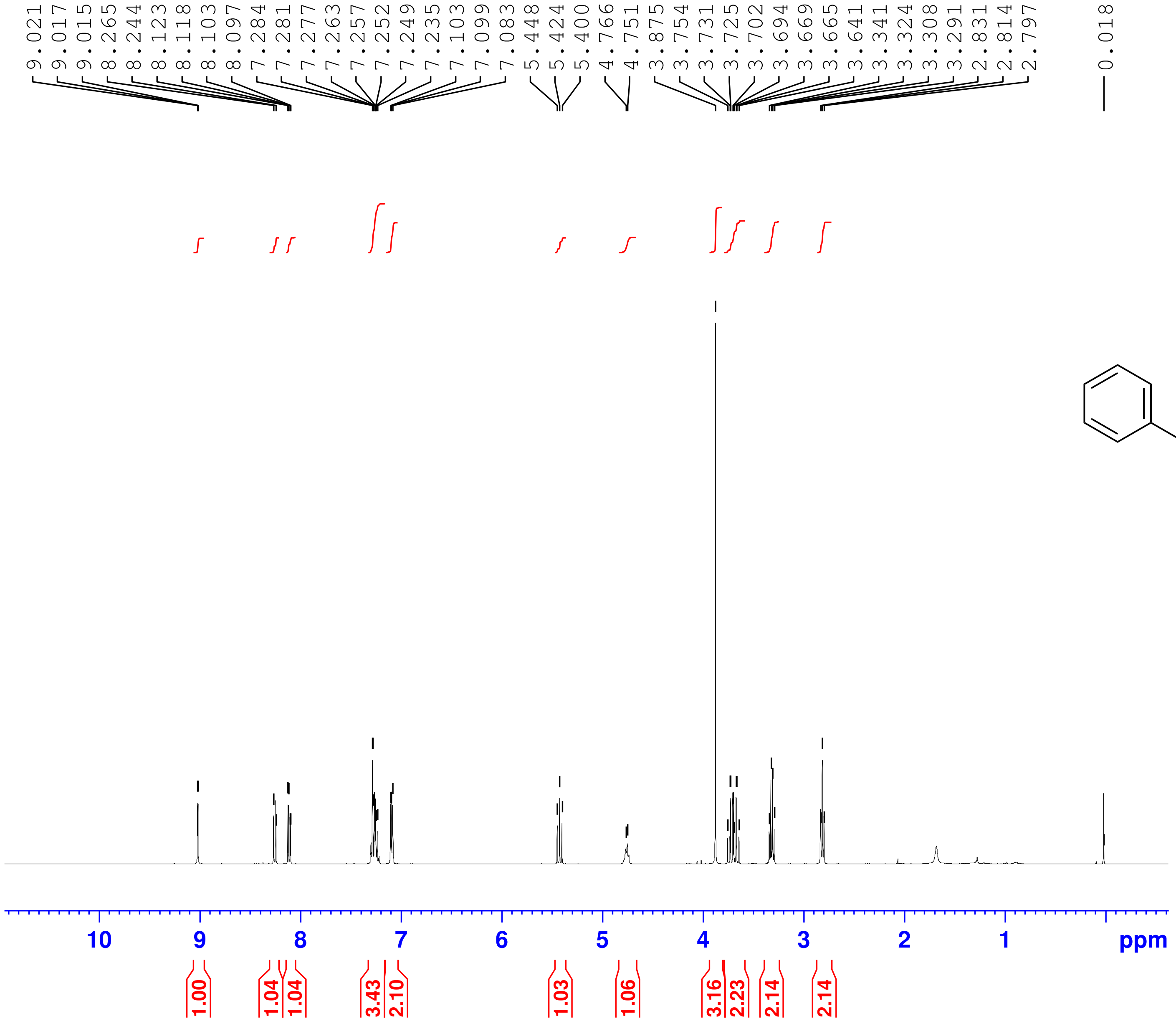

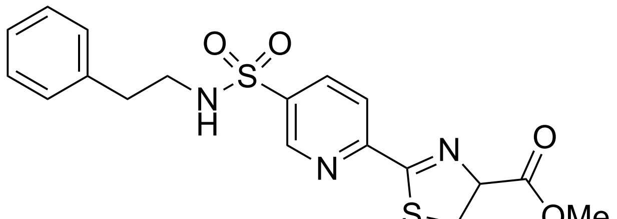

3h

^{13}C-NMR of **3h** (100 MHz, $CDCl_3$)

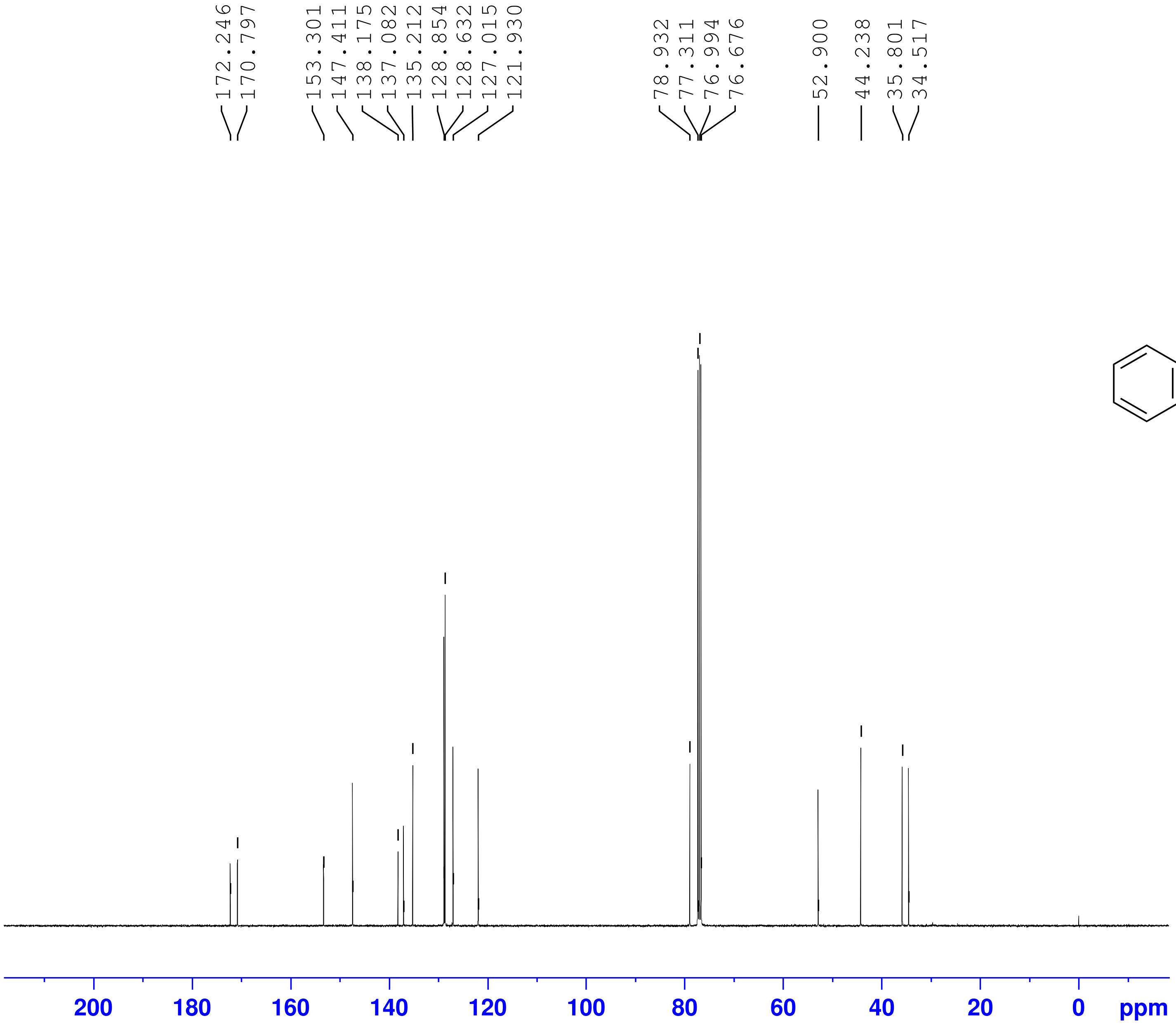

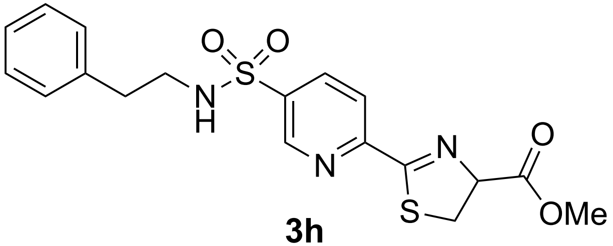

3h

^{1}H-NMR of **3i** (400 MHz, $CDCl_3$)

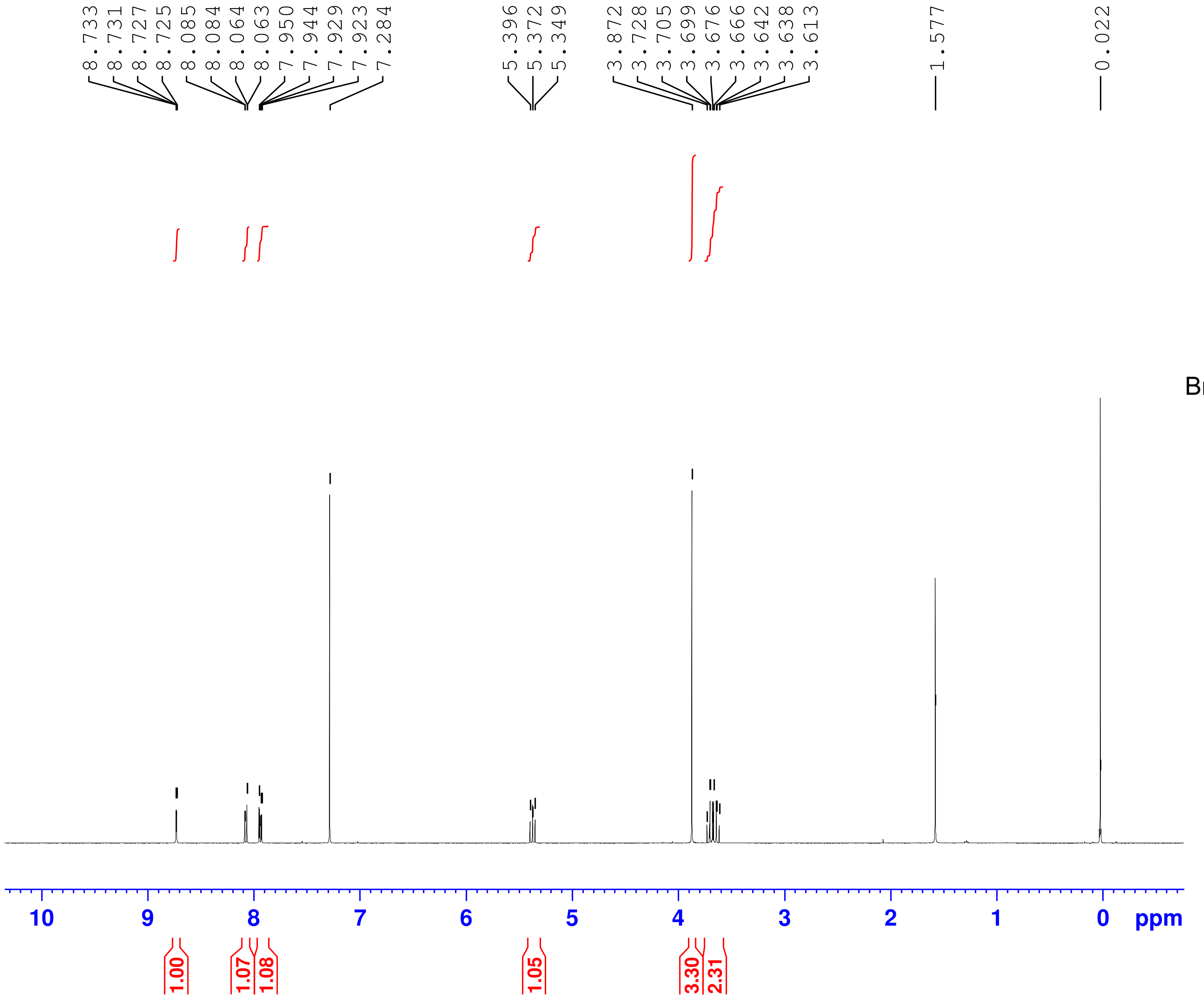

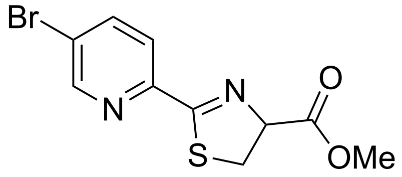

3i

^{13}C-NMR of **3i** (100 MHz, $CDCl_3$)

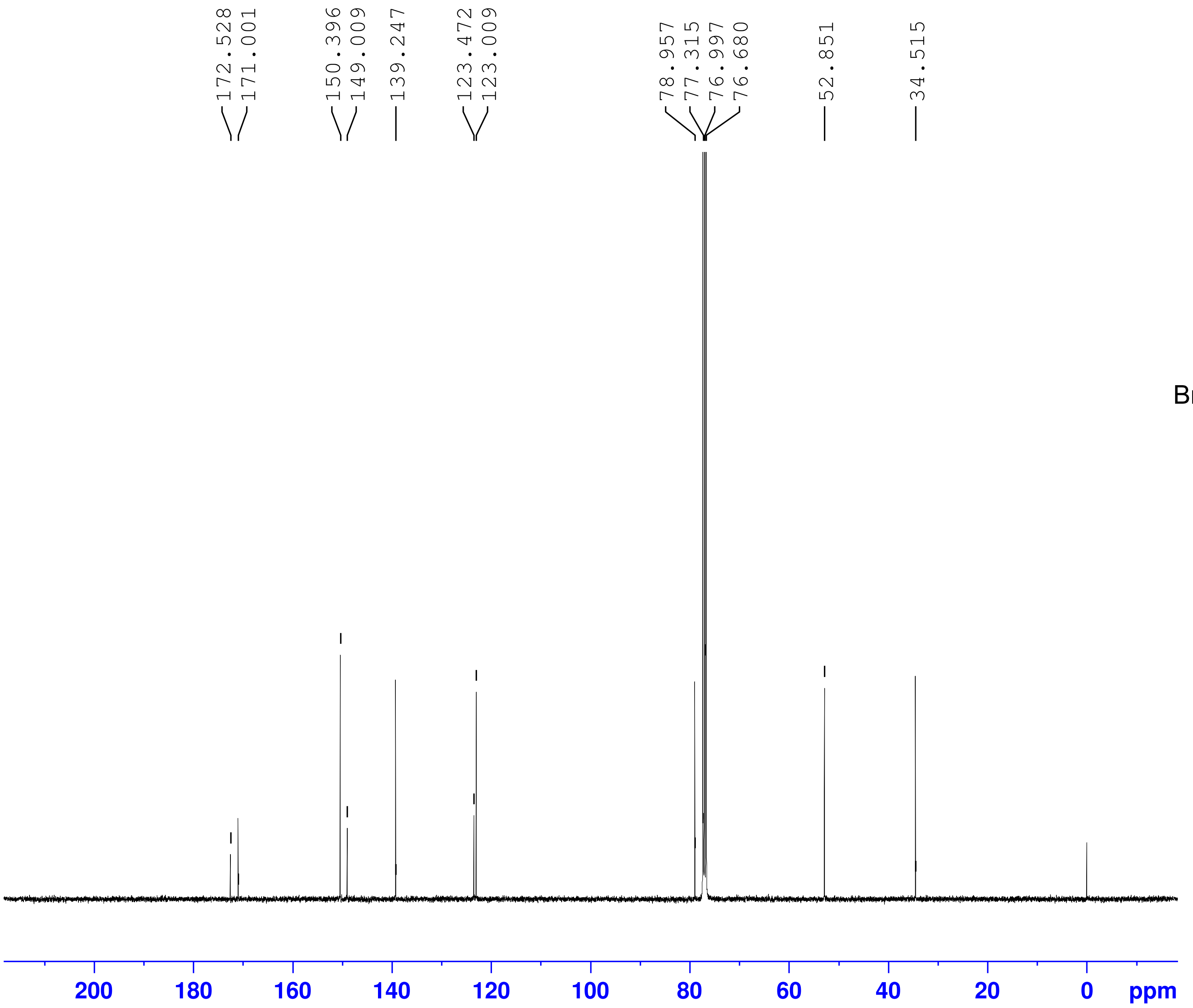

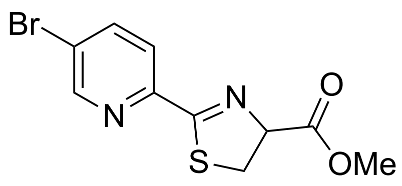

3i

^{1}H-NMR of **3j** (400 MHz, $CDCl_3$)

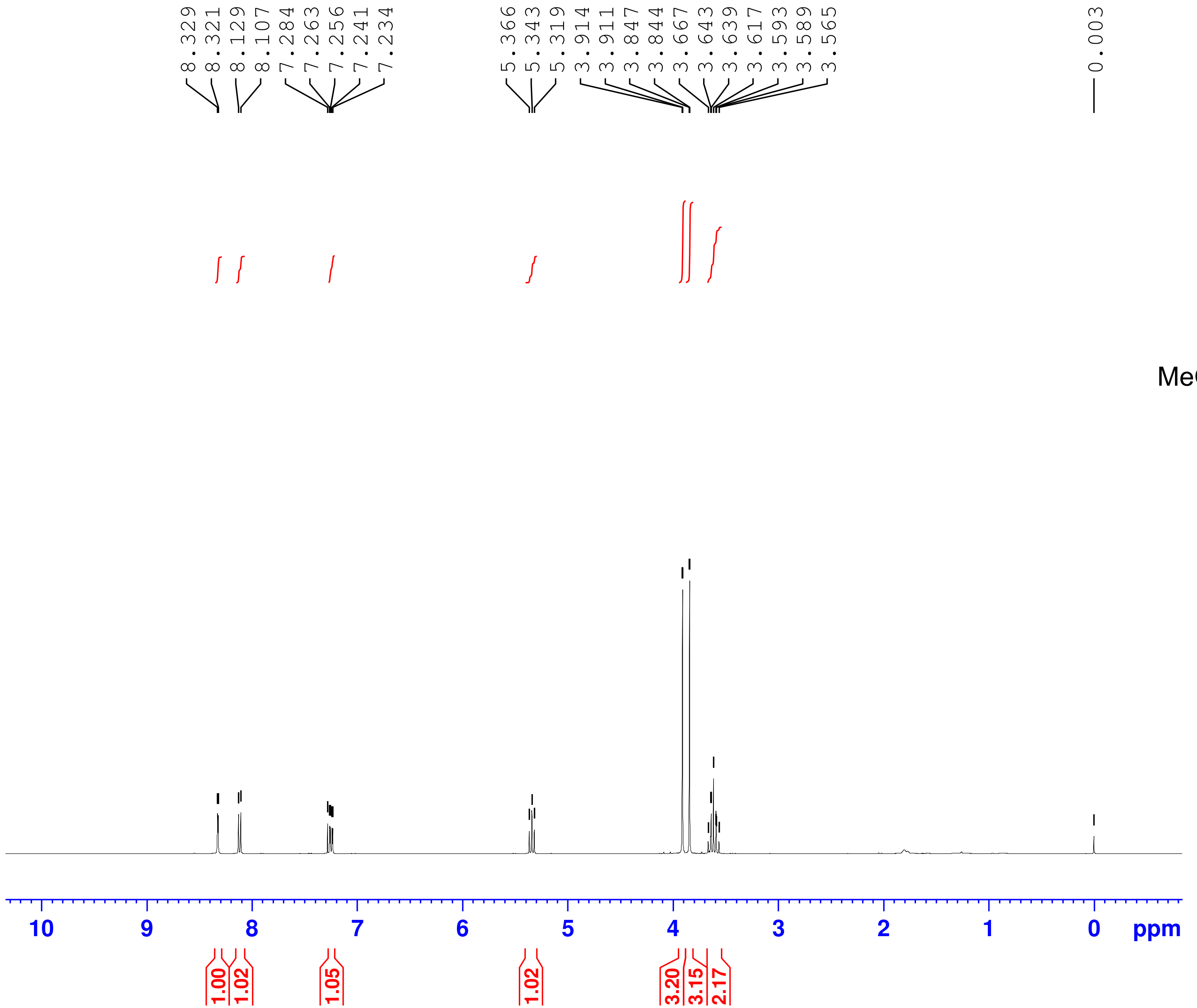

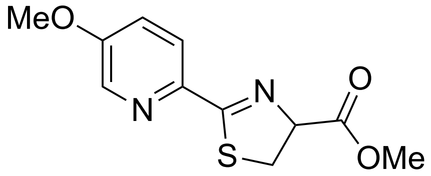

3j

^{13}C-NMR of **3j** (100 MHz, $CDCl_3$)

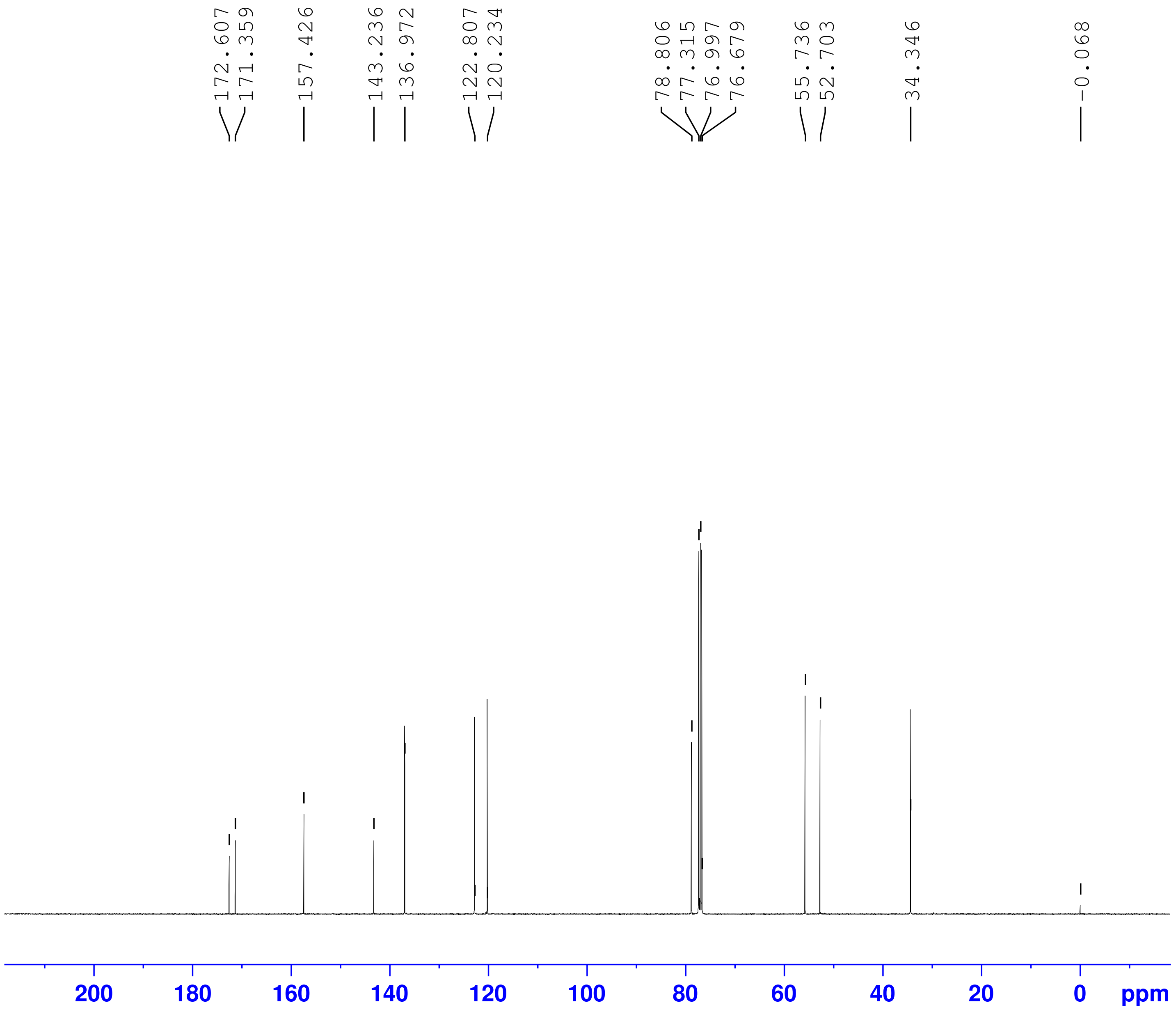

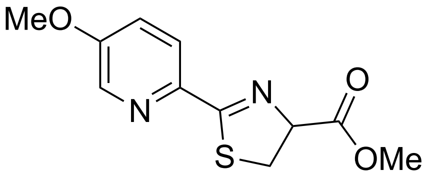

3j

^{1}H-NMR of **3k** (400 MHz, $CDCl_3$)

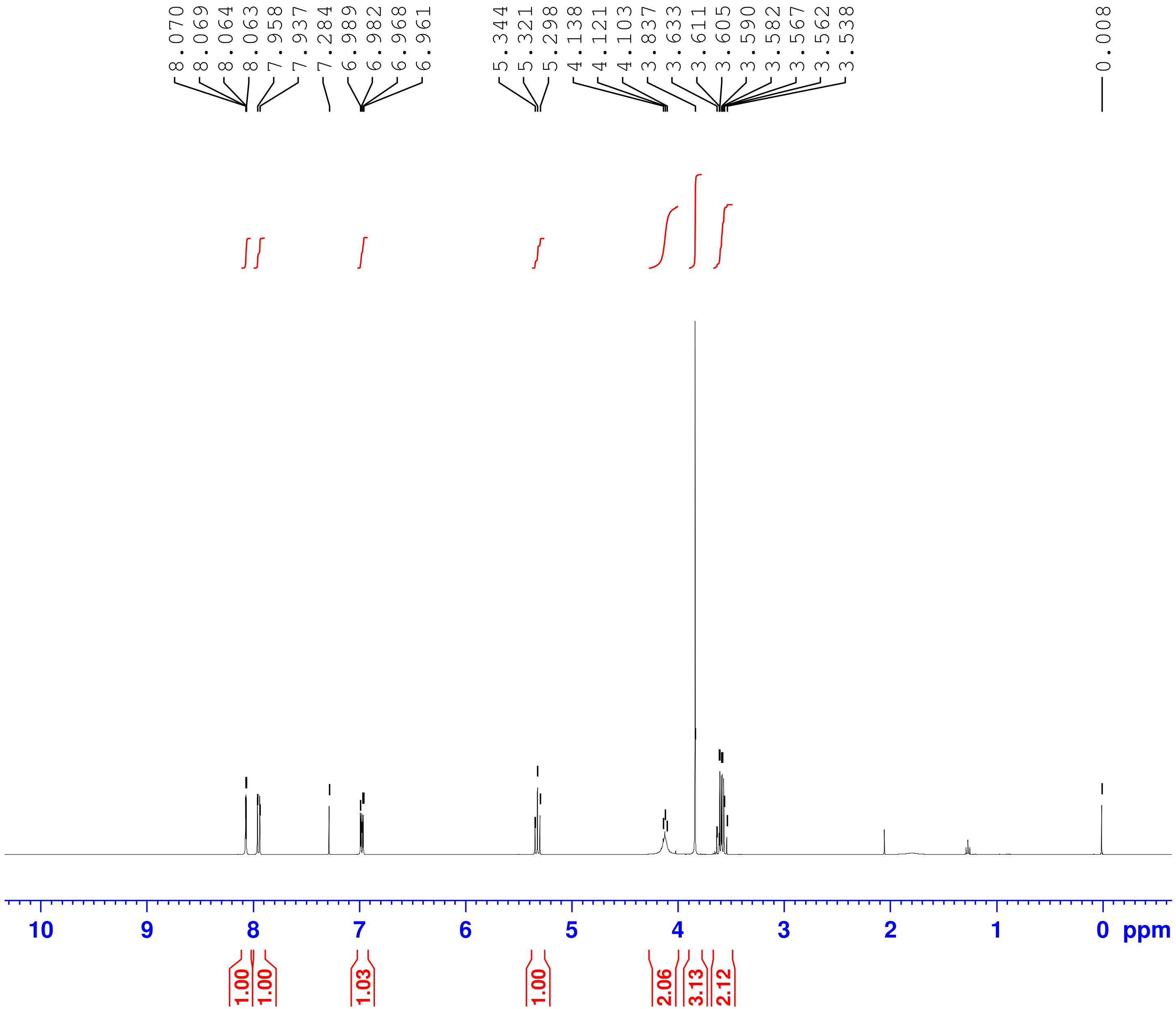

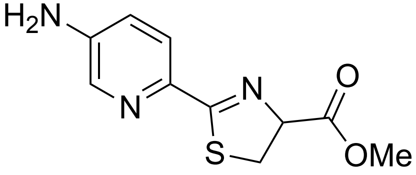

3k

^{13}C-NMR of **3k** (100 MHz, $CDCl_3$)

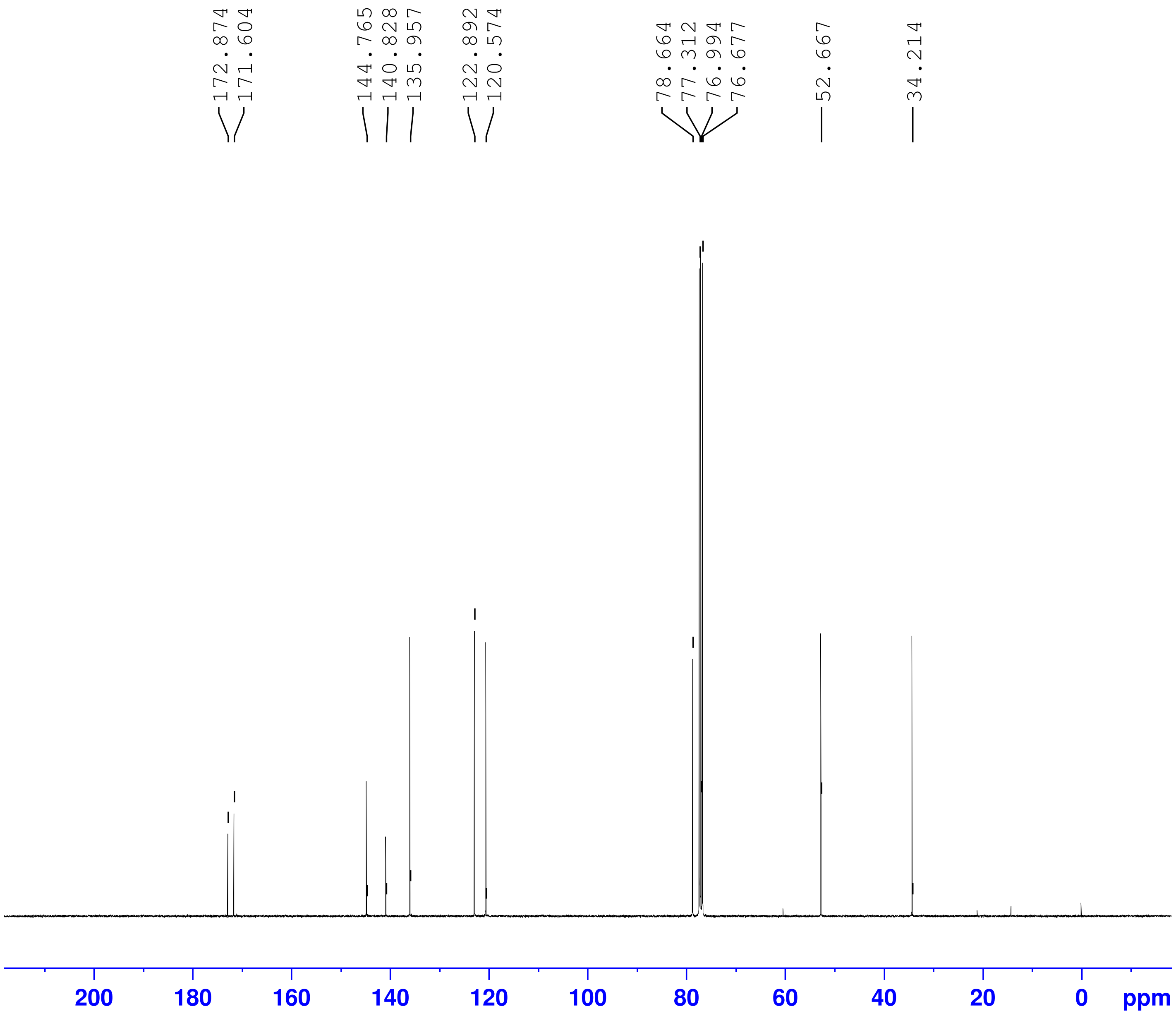

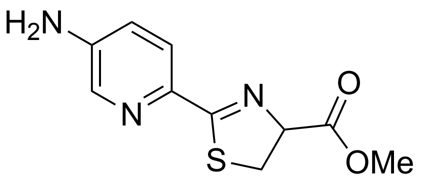

3k

^{1}H-NMR of **3l** (400 MHz, $CDCl_3$)

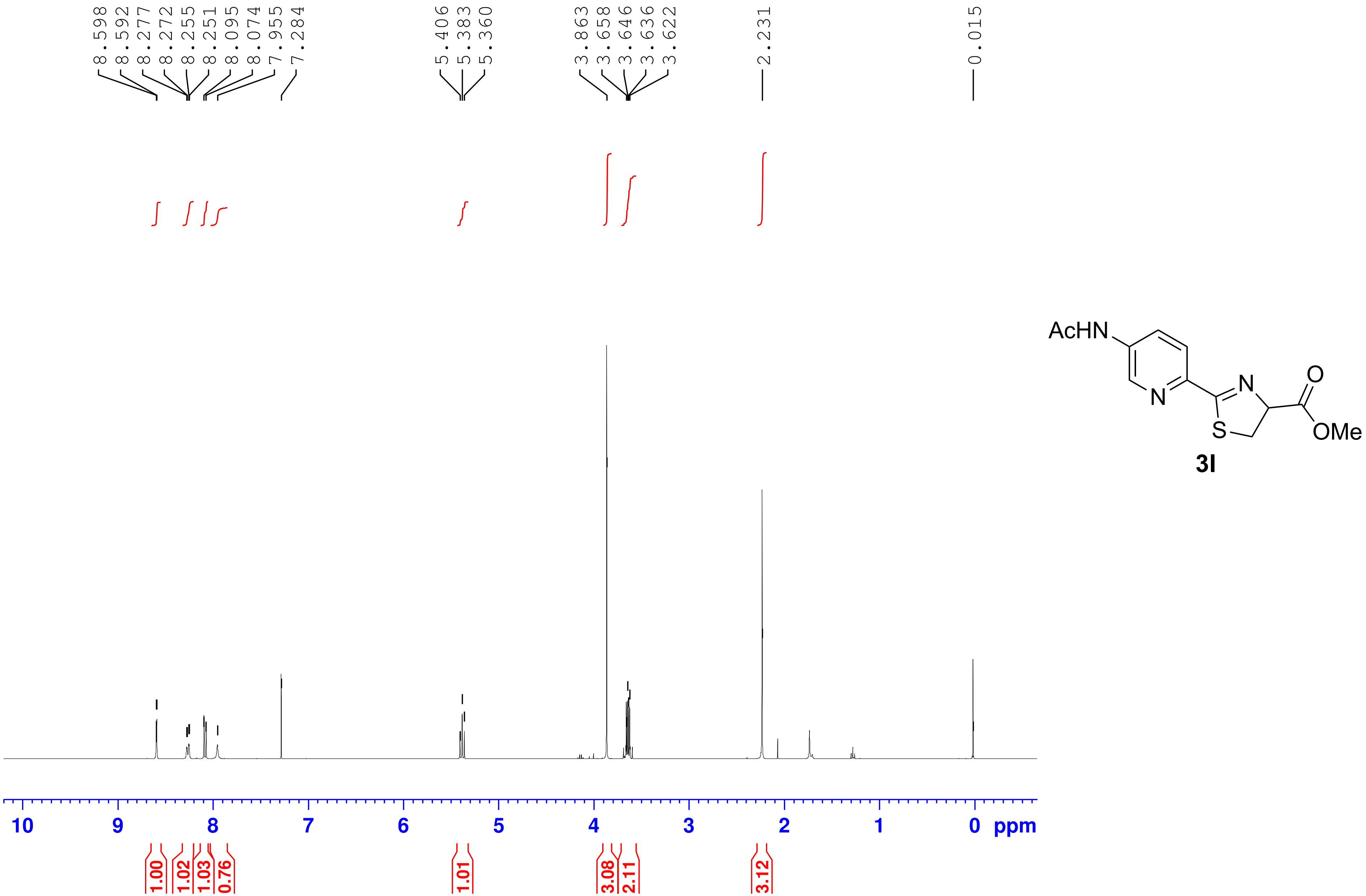

3l

^{13}C-NMR of **3l** (100 MHz, $CDCl_3$)

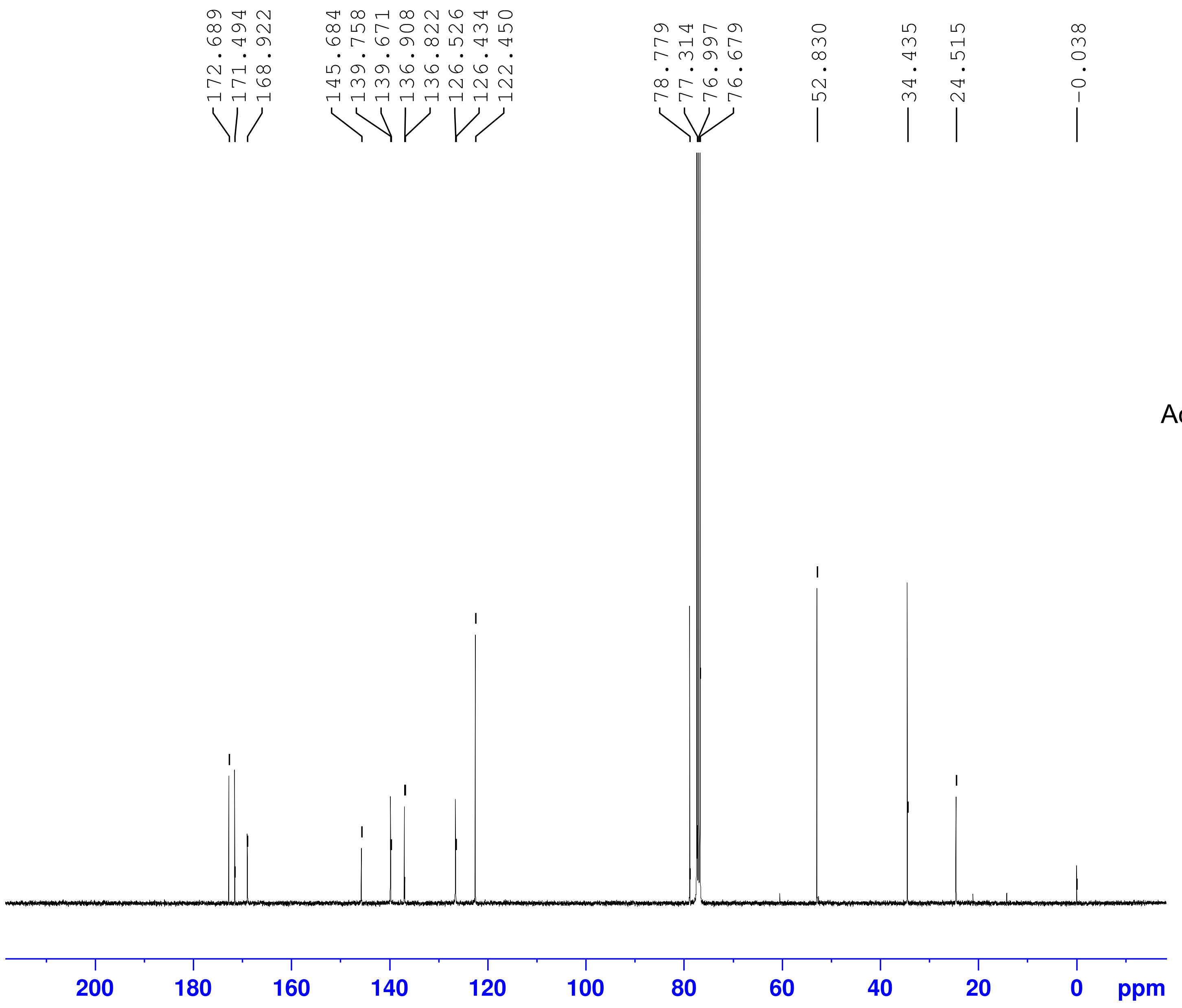

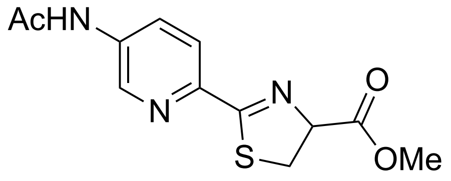

3l
